# Deformation analysis of lipid membranes subjected to general forms of intra-membrane viscous flow and interactions with an elliptical-cross-section substrate

**DOI:** 10.1038/s41598-019-57179-z

**Published:** 2020-01-16

**Authors:** Zhe Liu, Chun-il Kim

**Affiliations:** Department of Mechanical Engineering, University of Alberta, Edmonton, Alberta T6G 2G8 Canada

**Keywords:** Biomaterials - cells, Applied mathematics

## Abstract

We study the morphology of lipid membranes subjected to intra-membrane viscous flows and interactions with elliptical cylinder substrates. From the non-linear theory of elastic surfaces, a linearized shape equation and admissible boundary conditions are formulated in elliptical coordinates via the Monge representation of a surface. In particular, the intra-membrane viscosity terms are linearized and mapped into elliptic coordinates in order to accommodate more general forms of viscous flow. The assimilated viscous flow is characterized by potential functions which satisfies the continuity condition. A complete solution in terms of Mathieu function is then obtained within the prescription of incremental deformations superposed on large. The results describe smooth morphological transitions over the domain of interest and, more importantly, predicts wrinkle formations in the presence of intra-membrane viscous flow in the surface. Lastly, the obtained solution accommodates the results from the circular cases in the limit of vanishing eccentricity and intra-membrane viscous flow.

## Introduction

The mechanics of lipid membranes has consistently been the subject of intense research for its importance in the understanding of a wide variety of essential cellular processes^1–5^. Traditionally, it was believed that cells are surrounded by a thin oil-based barrier, yet the structure of this membrane was not well understood. In 1920s, E. Gorter and F. Grendel.^6^ found that the cell membrane is composed of lipid molecules (phospholipids) which are generally divided into two important groups: the hydrophilic head parts and hydrophobic tail groups. When dispersed into aqueous solutions, lipid molecules are driven by hydrophobic effects to form a unique bilayer structure (a lipid bilayer) with opposing orientations, that maintain symmetry about a mid-surface. In fact, with the advances in electron microscopy, the bilayer structure was identified as a characteristic of all biological membranes (biomembranes)^7^. Since they are negligibly thin (typically 5–10 nm), and fragile, the study of various aspects of lipid bilayers is often achieved by employing mathematical models in order to overcome the formidable difficulties of experimental studies. Also, from a mechanical perspective, the responses of a lipid membrane can be idealized as a thin elastic film. Within this context, the development of theoretical models describing the behavior of lipid bilayers has greatly benefited from the differential geometry of a surface and the theory of an elastic surface such that the deformation of energy of a thin membrane can be expressed by the mean and Gaussian curvatures of a surface^8,9^. In particular, Helfrich proposed a well-known Helfrich energy potential^1^ which addresses the symmetry of lipid bilayers and further ensures the resulting equilibrium state to be energy minimizing^10^. This, together with the use of variational principle, and the virtual-work statement, furnish the Euler-Lagrange equations also known as “membrane shape equations”, which have been successfully adopted in a wide range of problems (see, for example^11–13^). The variations of the classical Helfrich model have been continuously investigated in order to provide more efficient descriptions of lipid membranes’ morphology induce by various cellular activities, such as distensions^12^, tilts^14^, buddings^15,16^, spontaneous curvatures^11^ and substrate interactions^17–19^.

The study of the mechanical responses of membranes under the influences of intra-membrane viscous flow are of particular mechanical interest due to its importance in the explanation of essential cellular functions including budding, fission and vesicle formations^20–23^. The theoretical frame work accounting for the effects of intra-membrane viscosity into the model of membrane deformations has been established in^24^. In there, authors reveal that the dynamics of the membrane system is notably influenced by the presence of intra-membrane viscous flow. The authors in^25^ developed the comprehensive non-linear model of membranes incorporating the effects of intra-membrane viscosity from the elastic model of surfaces^26,27^. To this end, authors in^28^ discussed a compatible linear model within the setting of superposed incremental deformations. However, the analysis presented in^28^ is limited to certain types of problems where viscous flow is characterized as either constant or simple linear functions, and the interaction occurs through a circular contact region to obtain a mathematically tractable system. In a typical environment, a lipid membrane system is involved in more complex processes^4,5^ (e.g. interactions through a non-circular domain and the influences from multi-source viscous flows). Therefore, the development of a more comprehensive model may be necessary to promote researches on the related subjects.

In the present work, we study the deformations of lipid membranes interacting with intra-membrane viscous flow and an elliptical cylinder substrate. Utilizing the Monge parameterization of a surface and general curvilinear coordinates, the expressions of linearized shape equation and associated boundary conditions are obtained from the non-liner theory^25^. The intra-membrane viscosity terms are formulated by means of ‘admissible linearization’ and successively transformed into elliptical coordinates to assimilate more general types of viscous flow. More importantly, we obtained a complete analytic solution by employing adapted iterative reduction and the method of eigenfunction expansion^29–31^, which describes the deformations of lipid membranes when interacting with intra-membrane viscous flow and an elliptical-cross-section substrate. It is found that intra-membrane viscosity induces wrinkle formations of the lipid membrane and the corresponding number of wrinkles exhibits sensitivity to both the radius of the ellipse and the intensity of viscous flow. Comparisons with phenomenologically compatible cases such as a circular substrate -membrane interactions and capillary wrinkle of polymer films, are made where the proposed model successfully reproduces the results from^28,32^ in the limit of vanishing eccentricity of an ellipse. Further, we obtain solutions corresponding to the case of a lipid membrane subjected to non-uniform viscous flows and dual source flows. This is facilitated by the relaxed form of the prescribed tangential and normal force, and the condition of continuity along and within the elliptical boundaries, unlike those arising in circular boundaries where the admissible set of viscous flows are strictly uniform in one of the coordinate directions^28^. The resulting deformation fields show clear signs of dual source interference in that both the radial and circumferential wave forms are simultaneously predicted. Case study vis a vis morphologically similar results of shape memory films^33^ are presented to investigate the potential applicability of the proposed model in the analysis of different types of membrane. In particular, it is found that the principles of superposition from linear elasticity remains valid, even in the presence of general forms of dual source viscous potentials. That is the solution of a dual source problem can be directly obtained by adding solutions of two single source problems. The solutions presented here are of more practical interest in that, essentially, they lead to solutions of problems in which the viscosity effects are characterized by a wide class of potential functions and so can accommodate a correspondingly large set of physically relevant problems. For example, potential applications may be expected in the study of wrinkle-caused disease (e.g. a macular epiretinal membrane^34^) and the influences of membrane viscosity on various cellular functions such as fusion, fission and budding^35^. Further, the presented solution reproduces the existing results^17^ when viscosity effects are removed, and does incorporate the solution of the classical membrane-substrate interaction problem^11^ in the limit of vanishing eccentricity. In fact, the classical solution obtained directly from the proposed model produces more accurate predictions by identifying the additional Bessel functions, which is reduced from the Mathieu potentials.

Throughout the paper, we make use of a number of well-established symbols and conventions. Thus, unless otherwise stated, Greek indices take the values in {$1,2$} and, when repeated, are summed over their ranges. Lastly, ${(\ast )}_{,\alpha }$ denotes the derivative of ‘$\ast$’ with respect to a coordinate ${\theta }^{\alpha }$ and ${W}_{K}$ stands for the derivatives of a scalar-valued function $W(K)$ with respect to the parameter $k$.

## Viscous Lipid Membranes

In the study of the mechanics of lipid bilayer membranes, it is widely accepted that lipid membranes can be regarded as a continuous elastic surface. Within this idealization, the mechanical responses of a lipid membrane can be modeled via the theory of an elastic surface. In this section, we reformulate the results in the present context for the sake of clarity and completeness, and for the use in the derivation of the compatible linear model. The original derivation concerning the viscous elastic membranes can be found in^25^.

The equilibrium state of a purely elastic surface, in the presence of normal pressure $p$, is given by^26^

1 $${{\bf{T}}}_{;\alpha }^{\alpha }+p{\bf{n}}=0.$$

Here, ${{\bf{T}}}^{\alpha }$ and ${\bf{n}}$ are the *stress vectors* and the local surface unit normal, respectively, and the semi-colon denotes the surface covariant differentiation in the sense of the Levi-Civita connection on a surface. The associated surface metric is defined as

2 $${a}_{\alpha \beta }={{\bf{a}}}_{\alpha }\cdot {{\bf{a}}}_{\beta },$$

where ${{\bf{a}}}_{\alpha }={{\bf{r}}}_{,\alpha }({\theta }^{\alpha },t)$ are the tangent vectors to the surface $\omega$ induced by the parameterization ${\bf{r}}({\theta }^{\alpha },t),$ the position in ${{\mathbb{R}}}^{3}$ of a point on the surface with coordinate ${\theta }^{\alpha }$. The unit vector field **n**, which serves as the local surface orientation, is then computed as ${\bf{n}}{\boldsymbol{(}}{\theta }^{\alpha })=\tfrac{1}{2}{\varepsilon }^{\alpha \beta }{{\bf{a}}}_{\alpha }\times {{\bf{a}}}_{\beta }$, where ${\varepsilon }^{\alpha \beta }={e}^{\alpha \beta }/\sqrt{a}$ refers to the permutation tensor density with $a={\rm{\det }}({a}_{\alpha \beta })$ and ${e}^{11}={e}^{22}=0,{e}^{12}=-\,{e}^{21}=1$. The matrix of the surface metric is a positive-definite (i.e. $a > 0$), which further suggests the existence of dual metric ${a}^{\alpha \beta }$, the inverse of the metric ${a}_{\alpha \beta }$ (i.e. ${a}^{\alpha \beta }={({a}_{\alpha \beta })}^{-1}$). Thus, the dual basis (contravariant basis) can defined as ${{\bf{a}}}^{\alpha }={a}^{\alpha \beta }{{\bf{a}}}_{\beta }$. Combining the above results, the covariant differentiation of the surface covariant is then computed as^36^

3 $${{\bf{a}}}_{\alpha ;\beta }={{\bf{a}}}_{\alpha ,\beta }-{\Gamma }_{\alpha \beta }^{\lambda }{{\bf{a}}}_{\lambda },$$

where ${\Gamma }_{\alpha \beta }^{\lambda }={{\bf{a}}}_{\alpha ,\beta }\cdot {{\bf{a}}}^{\lambda }$ are the Christoffel symbols induced by the local surface coordinate. These results furnish the well-known Gauss and Weingarten equations:

4 $${{\bf{a}}}_{\alpha ,\beta }=({{\bf{a}}}_{\alpha ,\beta }\cdot {{\bf{a}}}^{\lambda }){{\bf{a}}}_{\gamma }+{\boldsymbol{(}}{{\bf{a}}}_{\alpha ,\beta }\cdot {\bf{n}}){\bf{n}}={\Gamma }_{\alpha \beta }^{\lambda }{{\bf{a}}}_{\gamma }+{b}_{\alpha \beta }{\bf{n}};{b}_{\alpha \beta }={{\bf{a}}}_{\alpha ,\beta }\cdot {\bf{n}}({\rm{Gauss}}),\,{\rm{and}}$$

5 $${b}_{\alpha \beta }=-\,{{\bf{n}}}_{,\alpha }\cdot {{\bf{a}}}_{\beta }({\rm{Weingarten}}),$$

where ${b}_{\alpha \beta }$ are the coefficients of the second fundamental form of surface and its covariant cofactor is defined by

6 $${\tilde{b}}^{\alpha \beta }={\varepsilon }^{\alpha \lambda }{\varepsilon }^{\beta \gamma }{b}_{\lambda \gamma }.$$

The deformation energy of an elastic surface can be expressed via the above two primary parameters: the coefficient of the first fundamental form ${a}_{\alpha \beta }$ and the second fundamental form ${b}_{\alpha \beta }$^8,9,26^. Thus, for example, an elastic surface whose free-energy density is expressed by the mean and Gaussian curvatures through ${a}_{\alpha \beta }$ and ${b}_{\alpha \beta }$ (i.e. $W=W(H,K,\rho ;{a}_{\alpha \beta },{b}_{\alpha \beta })$), ${{\bf{T}}}^{\alpha }$ takes the following compact form^26^:

7 $${{\bf{T}}}^{\alpha }=({\sigma }^{\beta \alpha }+{b}_{\mu }^{\beta }{M}^{\mu \alpha }){{\bf{a}}}_{\beta }-{M}_{;\beta }^{\alpha \beta }{\bf{n}},$$

where

8 $${\sigma }^{\beta \alpha }=(\lambda +W){a}^{\alpha \beta }-\mathrm{(2}{W}_{H}H+2{W}_{K}K){a}^{\alpha \beta }+{W}_{H}{\tilde{b}}^{\alpha \beta },$$

9 $${M}^{\beta \alpha }=\frac{1}{2}{W}_{H}{a}^{\alpha \beta }+{W}_{K}{\tilde{b}}^{\alpha \beta }.$$

and $\lambda$ is the constitutively-indeterminate Lagrange-multiplier field. The corresponding mean and Gaussian curvatures are computed as^36^

10 $$H=\tfrac{1}{2}{a}^{\alpha \beta }{b}_{\alpha \beta }\,{\rm{and}}\,K=\tfrac{1}{2}{\varepsilon }^{\alpha \beta }{\varepsilon }^{\lambda \mu }{b}_{\alpha \lambda }{b}_{\beta \mu },$$

which also satisfy the following equalities

11 $${b}^{\alpha \beta }=2H{a}^{\alpha \beta }-{\tilde{b}}^{\alpha \beta }(\text{Cayley}\,-\,{\rm{Hamilton}}\,{\rm{theorem}})\,{\rm{and}}\,{a}^{\beta \alpha }K={b}_{\mu }^{\beta }{\tilde{b}}^{\mu \alpha }.$$

Now, the viscous stress induced by the straining effects of the fluid is given by^37^

12 $${\sigma }^{\alpha \beta }=(\lambda +W){a}^{\alpha \beta }+\nu {a}^{\alpha \lambda }{a}^{\beta \mu }{\dot{a}}_{\lambda \mu },$$

where $\nu$ is the intra-membrane shear viscosity and

13 $${\dot{a}}_{\lambda \mu }\mathop{=}\limits^{({\rm{Eq}}\mathrm{.2)}}({{\bf{a}}}_{\lambda }\cdot {{\bf{a}}}_{\mu }\dot{)}={\dot{{\bf{a}}}}_{\lambda }\cdot {{\bf{a}}}_{\mu }+{{\bf{a}}}_{\lambda }\cdot {\dot{{\bf{a}}}}_{\mu }$$

is the time derivative of the evolving surface metric. Thus, in order to compute viscous stress, it is required to compute ${\dot{{\bf{a}}}}_{\mu }$, which can be obtained via the material time derivative of a position vector ${\bf{r}}$^26^:

14 $${\bf{u}}=\dot{{\bf{r}}}=\frac{\partial {\bf{r}}}{\partial t}+\frac{\partial {\bf{r}}}{\partial {\theta }^{\alpha }}\frac{\partial {\theta }^{\alpha }}{\partial t}={{\bf{r}}}_{t}+{{\bf{a}}}_{\alpha }{v}^{\alpha }.$$

Accordingly, it is found that

15 $$\begin{array}{rcl}{\dot{{\bf{a}}}}_{\lambda } & = & {{\bf{u}}}_{,\lambda }={({v}^{\alpha }{{\bf{a}}}_{\alpha }+w{\bf{n}})}_{,\lambda }={v}_{,\lambda }^{\alpha }{{\bf{a}}}_{\alpha }+{v}^{\alpha }{{\bf{a}}}_{\alpha ,\lambda }+{w}_{,\lambda }{\bf{n}}+w{{\bf{n}}}_{,\lambda }\\  & = & ({v}_{\alpha ;\lambda }-w{b}_{\alpha \lambda }){{\bf{a}}}^{\alpha }+({v}^{\alpha }{b}_{\alpha \lambda }+{w}_{,\lambda }){\bf{n}}{\boldsymbol{.}}\,\,\because {v}_{\alpha ;\beta }={v}_{\alpha ,\beta }-{v}_{\beta }{\Gamma }_{\alpha \beta }^{\lambda },\end{array}$$

and

16 $${\dot{a}}_{\lambda \mu }\mathop{=}\limits^{({\rm{Eqs}}.\,13\,\& \,15)}{v}_{\mu ;\lambda }+{v}_{\lambda ;\mu }-2w{b}_{\lambda \mu },$$

where ${v}^{\alpha }=\partial {\theta }^{\alpha }/\partial t,$ and ${{\bf{r}}}_{t}=|{{\bf{r}}}_{t}|{\bf{n}}=w{\bf{n}}$ are respectively the tangential and normal velocities of a material point on the initial surface^26,37,38^.

It is now straightforward to show from Eqs. (8), (12) and (16) that,

17 $$\begin{array}{rcl}{\sigma }^{\beta \alpha } & = & (\lambda +W){a}^{\beta \alpha }-\mathrm{2(}{W}_{H}H+{W}_{K}K){a}^{\beta \alpha }+{W}_{H}{\tilde{b}}^{\beta \alpha }\\  &  & +\,\nu [{a}^{\beta \lambda }{a}^{\alpha \mu }({v}_{\mu ;\lambda }+{v}_{\lambda ;\mu })-4wH{a}^{\beta \alpha }+2w{\tilde{b}}^{\beta \alpha }],\end{array}$$

which is the expression of the viscous stress.

Thus, by means of Eqs. (9), (11)_2_ and (17), and applying the conventional Euclidean dot product in normal ${\bf{n}}$ direction, Eq. (1) becomes^25^

18 $$\begin{array}{rcl}p & = & {W}_{H}\mathrm{(2}{H}^{2}-K)+2H({W}_{K}K-W)-2\lambda H\\  &  & +\,\Delta (\frac{1}{2}{W}_{H})+{({W}_{K})}_{;\alpha \beta }{\tilde{b}}^{\alpha \beta }\\  &  & -\,2\nu [\frac{1}{2}{b}^{\alpha \beta }({v}_{\alpha ;\beta }+{v}_{\beta ;\alpha })-2w\mathrm{(2}{H}^{2}-K)],\end{array}$$

which serves as the equation of motion (normal direction) of the lipid membrane in the presence of intra-membrane viscosity effects. Further, Δ is the Laplace-Beltrami operator (i.e. $\Delta \phi ={\phi }_{;\alpha \beta }{a}^{\alpha \beta }$) on the surface $\Omega$. Consequently, by projecting Eq. (1) onto the basis coordinate plane of ${{\bf{a}}}_{\alpha }$, the following tangential equations of motion can be obtained:

19 $${\lambda }_{,\alpha }-4vw{H}_{,\alpha }+2\nu [\frac{1}{2}{a}^{\lambda \mu }{({v}_{\mu ;\alpha }+{v}_{\alpha ;\mu })}_{;\lambda }-{w}_{,\lambda }{b}_{\alpha }^{\lambda }]=0.$$

Much of literature on the mechanics of lipid membranes has revealed that a bilayer membrane can be regarded as a continuous two-dimensional elastic surface where the response functions are governed by the well-known Helfrich energy potential^1^. The model has been widely adopted in various subjects within bilayer membrane mechanics (see, for example^11,12,15^, and the references therein). Following the work of^25^, in this paper, we consider a symmetric membrane of Helfich type (i.e. $W(H,K)=W(\,-\,H,K)$), subjected to the membrane-substrate interactions and the effects of intra-membrane viscosity. The corresponding free-energy density function is defined by

20 $$W=k{H}^{2}+\bar{k}K,$$

where $k$ and $\bar{k}$ are empirical bending constants, which pertain to lipid membranes with uniform properties. Thus, from Eqs. (18) and (20), becomes

21 $$p=k[\Delta H+2H({H}^{2}-K)]-2\lambda H-2\nu [\frac{1}{2}{b}^{\alpha \beta }({v}_{\alpha ;\beta }+{v}_{\beta ;\alpha })-2w\mathrm{(2}{H}^{2}-K)],$$

while the tangential equations (Eq. (19)) remain intact.

Lastly, by invoking Eq. (16), the condition of an incompressible fluid $\dot{J}/J=\frac{1}{2}{a}^{\alpha \beta }{\dot{a}}_{\alpha \beta }=0$ can be obtained as^39^

22 $${v}_{;\alpha }^{\alpha }-2wH=0,$$

where $$H=\tfrac{1}{2}{a}^{\alpha \beta }{b}_{\alpha \beta }$$.

## Incremental Deformations of Lipid Membranes

The use of Monge parameterization and admissible linearization is a widely adopted methodology for lipid membrane analysis, and the associated procedures are well documented in the literature (see, for example^11,15,18^). Here, we reformulate the results for the sake of completeness. Under the Monge parameterization, material points on the membrane surface $\Omega$ is defined by

23 $${\bf{r}}({\theta }^{\alpha },t)={\boldsymbol{\theta }}+z({\boldsymbol{\theta }},t){\bf{k}},$$

where ${\boldsymbol{\theta }}({\theta }^{\alpha })$ is position on a plane $p$ with unit normal **k**. The problem of determining the membranes’ deformed configuration is then reduced to solving a single function $z({\boldsymbol{\theta }},t)$. In the cases of Cartesian coordinates, we have

24 $${\boldsymbol{\theta }}={\theta }^{\alpha }{{\bf{e}}}_{\alpha },$$

where $\{{{\bf{e}}}_{\alpha }\}$ is an orthonormal basis for the plane and, the subscripts of the surface coordinates are dropped and replaced by $1=x,2=y$, unless otherwise specified. Accordingly, we compute

25 $$\begin{array}{rcl}{{\bf{r}}}_{,t} & = & {z}_{,t}{\bf{k}},\,{{\bf{a}}}_{\alpha }={{\bf{e}}}_{\alpha }+{z}_{,\alpha }{\bf{k}},\,a=det({a}_{\alpha \beta })=[1+{(z{,}_{1})}^{2}+{(z{,}_{2})}^{2}],\\ {a}^{\alpha \beta } & = & {\delta }_{\alpha \beta }+{z}_{,\alpha }{z}_{,\beta },\\ H & = & \frac{(1+{z}_{\mathrm{,2}}^{2}){z}_{\mathrm{,11}}+(1+{z}_{\mathrm{,1}}^{2}){z}_{\mathrm{,22}}-2{z}_{\mathrm{,1}}{z}_{\mathrm{,2}}{z}_{\mathrm{,12}}}{2{a}^{\mathrm{3/2}}},\\ K & = & \frac{({z}_{\mathrm{,11}}{z}_{\mathrm{,22}}-{z}_{\mathrm{,12}}^{2})}{{a}^{2}},\\ {\bf{n}} & = & \frac{({\bf{k}}-\nabla z)}{\sqrt{a}}\,{\rm{and}}\,{\bf{b}}={b}_{\alpha \beta }({{\bf{a}}}^{\alpha }\otimes {{\bf{a}}}^{\beta })=\frac{{z}_{,\alpha \beta }}{\sqrt{a}}({{\bf{a}}}^{\alpha }\otimes {{\bf{a}}}^{\beta }\mathrm{)}.\end{array}$$

Here, $\nabla z={z}_{,\alpha }{{\bf{e}}}_{\alpha }$ is surface gradient, ${\delta }_{\alpha \beta }$ is Kronecker delta and ${\bf{b}}$ is the curvature tensor. Further, the expressions of the dual basis and the Christoffel symbols are obtained as

26 $$\begin{array}{rcl}{\Gamma }_{\alpha \beta }^{\lambda } & = & {z}_{,\lambda }{z}_{,\alpha \beta }/\sqrt{a},\\ {{\bf{a}}}^{1} & = & \frac{1}{a}[(1+{z}_{\mathrm{,2}}^{2})({{\bf{e}}}_{1}+{z}_{\mathrm{,1}}{\bf{k}})-{z}_{\mathrm{,1}}{z}_{\mathrm{,2}}({{\bf{e}}}_{2}+{z}_{\mathrm{,2}}{\bf{k}})],\,{\rm{and}}\\ {{\bf{a}}}^{2} & = & \frac{1}{a}[(1+{z}_{\mathrm{,1}}^{2})({{\bf{e}}}_{2}+{z}_{\mathrm{,2}}{\bf{k}})-{z}_{\mathrm{,1}}{z}_{\mathrm{,2}}({{\bf{e}}}_{1}+{z}_{\mathrm{,1}}{\bf{k}})].\end{array}$$

In the incremental deformation analysis, it is assumed that the gradient of $z({\theta }^{\alpha },t)$ of all orders are ‘small’ so that their products can be neglected. The procedure is commonly referred to as admissible linearization through which the geometrical and kinematical quantities associated with the surface (Eq. (19)) can be approximated as

27 $$\begin{array}{l}a\simeq 1,\,w\simeq {z}_{t},\,{\bf{n}}={\bf{k}}-{\nabla }_{p}z,\,{{\bf{a}}}^{\alpha }\simeq {{\bf{a}}}_{\alpha }={{\bf{e}}}_{\alpha }+{z}_{,\alpha }{\bf{k}},\,{\Gamma }_{\alpha \beta }^{\lambda }\simeq 0,\,{\bf{n}}\cdot {\bf{r}}\simeq z-{z}_{,\alpha }{\theta }^{\alpha },\\ {\bf{b}}\simeq {\nabla }_{p}^{2}z,\,H\simeq \frac{1}{2}{\Delta }_{p}z\,{\rm{and}}\,K\simeq \mathrm{0,}\end{array}$$

where the subscript ${(\ast )}_{p}$ refers to the projected counterparts of $(\ast )$ on the coordinate plane ${\omega }_{p}$, ${\nabla }_{p}^{2}z={z}_{,\alpha \beta }$ ${{\bf{e}}}_{\alpha }\otimes {{\bf{e}}}_{\beta }$ is the second gradient, and ${\Delta }_{p}z=tr({\nabla }_{p}^{2}z)$ is the corresponding Laplacian, respectively.

### Linearization of the intra-membrane viscosity terms

In the forthcoming derivations, we present the linearization procedures for the terms associated with the intra-surface viscous flow, which arise in the formulation of membrane equilibrium equations. To proceed, we express the surface gradient of the viscous flow fields and the curvature tensor as

28 $$\begin{array}{rcl}\nabla {\bf{v}} & = & \nabla ({v}_{\alpha }{{\bf{a}}}^{\alpha })=({v}_{\alpha ,\beta }{{\bf{a}}}^{\alpha }+{v}_{\alpha }{{\bf{a}}}_{,\beta }^{\alpha })\otimes {{\bf{a}}}^{\beta }\mathop{=}\limits^{({\rm{Eqs}}.3-\mathrm{4)}}{v}_{\alpha ;\beta }{{\bf{a}}}^{\alpha }\otimes {{\bf{a}}}^{\beta },\,{\rm{and}}\\ {\bf{b}} & = & {b}^{\alpha \beta }({{\bf{a}}}_{\alpha }\otimes {{\bf{a}}}_{\beta }\mathrm{)}.\end{array}$$

We then compute their traces to obtain

29 $$tr({\bf{b}}{(\nabla {\bf{v}})}^{T})+tr({\bf{b}}(\nabla {\bf{v}}))={b}^{\alpha \beta }({v}_{\alpha ;\beta }+{v}_{\beta ;\alpha }),$$

where ${{\bf{a}}}_{\alpha }\cdot {{\bf{a}}}^{\beta }={\delta }_{\alpha }^{\beta }$. Also, from the results in Eqs. (27) and (28) can be approximated, up to the leading order, to

30 $$\begin{array}{rcl}{v}_{\alpha ;\beta }({{\bf{a}}}^{\alpha }\otimes {{\bf{a}}}^{\beta }) & = & ({v}_{\alpha ,\beta }-{v}_{\lambda }{\Gamma }_{\alpha \beta }^{\lambda })[({{\bf{e}}}_{\alpha }+{z}_{,\alpha }{\bf{k}}{\boldsymbol{)}}\otimes ({{\bf{e}}}_{\beta }+{z}_{,\beta }{\bf{k}})]\\  & \simeq  & ({v}_{\alpha ,\beta })({{\bf{e}}}_{\alpha }\otimes {{\bf{e}}}_{\beta }),\,{\rm{and}}\\ {\bf{b}} & = & {b}_{\lambda \gamma }{a}^{\lambda \alpha }{a}^{\gamma \beta }({{\bf{a}}}_{\alpha }\otimes {{\bf{a}}}_{\beta })\\  & = & {b}_{\lambda \gamma }({\delta }_{\lambda \alpha }+{z}_{,\lambda }{z}_{,\alpha })({\delta }_{\gamma \beta }+{z}_{,\gamma }{z}_{,\alpha \beta })({{\bf{a}}}_{\alpha }\otimes {{\bf{a}}}_{\beta })\\  & \simeq  & {z}_{,\alpha \beta }{{\bf{e}}}_{\alpha }\otimes {{\bf{e}}}_{\beta }.\end{array}$$

Now, combining Eqs. (29) and (30), we find that,

31 $$tr({\bf{b}}{(\nabla {\bf{v}})}^{T})=[{v}_{\alpha ,\beta }({{\bf{e}}}_{\alpha }\otimes {{\bf{e}}}_{\beta }){z}_{,\lambda \gamma }({{\bf{e}}}_{\gamma }\otimes {{\bf{e}}}_{\lambda })]\simeq {v}_{\alpha ,\beta }{z}_{,\alpha \beta }\,{\rm{and}}\,tr({\bf{b}}(\nabla {\bf{v}}))\simeq {v}_{\beta ,\alpha }{z}_{,\alpha \beta }.$$

Thus, Eq. (29) simplifies to

32 $${b}^{\alpha \beta }({v}_{\alpha ;\beta }+{v}_{\beta ;\alpha })\simeq {z}_{,\alpha \beta }({v}_{\alpha ,\beta }+{v}_{\beta ,\alpha }\mathrm{)}.$$

However, since ${z}_{,\alpha \beta }={z}_{,\beta \alpha }$, the above can be re-written as

33 $${z}_{,\alpha \beta }({v}_{\alpha ,\beta }+{v}_{\beta ,\alpha })=2{z}_{,\alpha \beta }{v}_{\alpha ,\beta }.$$

To obtain the simplified expression of incompressibility condition (22), we evaluate the surface divergence of the viscous flow field as

34 $${\rm{div}}\,{\bf{v}}=tr(\nabla ({v}_{\alpha }{{\bf{a}}}^{\alpha }))\mathop{=}\limits^{({\rm{Eqs}}.\,\mathrm{28)}}tr[{v}_{\alpha ;\beta }{{\bf{a}}}^{\alpha }\otimes {{\bf{a}}}^{\beta }]\mathop{=}\limits^{({\rm{Eqs}}.\,\mathrm{2)}}{v}_{\alpha ;\beta }{a}^{\alpha \beta }={v}_{;\alpha }^{\alpha },$$

where ${{\bf{a}}}^{\alpha }\cdot {{\bf{a}}}^{\beta }={{\bf{a}}}_{\lambda }{a}^{\lambda \alpha }\cdot {{\bf{a}}}_{\gamma }{a}^{\lambda \alpha }={a}_{\lambda \gamma }{a}^{\lambda \alpha }{a}^{\lambda \alpha }={a}^{\alpha \beta }$. Substituting the above into Eq. (22), and further invoking Eq. (14), we arrive at

35 $${v}_{;\alpha }^{\alpha }-2wH={\rm{div}}\,{\bf{v}}-2(\frac{{z}_{,t}}{\sqrt{a}})H.$$

Thus, from Eq. (27), the leading order approximation of the above can be found as

36 $${v}_{;\alpha }^{\alpha }-2wH={({\rm{div}}{\bf{v}}{\boldsymbol{)}}}_{P}={v}_{\alpha ,a},$$

where ${({\rm{div}}{\bf{v}})}_{P}$ is the divergence of the projected coordinate plane ${\Omega }_{p}$.

Consequently, substitution of these linearized expressions, (Eqs. (27), (33) and (36)), into Eqs. (19, 21 and 22) delivers the following normal and tangential equations, and incompressibility conditions:

37 $$\frac{1}{2}k{\Delta }_{p}({\Delta }_{p}z)-\lambda {\Delta }_{p}z-2\nu {z}_{,\alpha \beta }{v}_{\alpha ,\beta }\simeq p,{(\lambda +P)}_{,\alpha }+v{\Delta }_{p}{v}_{\alpha }\simeq 0\,{\rm{and}}\,{v}_{\alpha ,\alpha }\simeq 0.$$

Here, $P=P({\theta }^{\alpha },t)$ is understood as a sequence of prescribed surface pressure (see^25^) from the admissible set of boundary forces which satisfy

38 $${f}_{\tau }=\nu ({v}_{\alpha ,\beta }+{v}_{\beta ,\alpha })({\tau }_{\beta }{\gamma }_{\alpha }{)}_{p}\simeq 0\,{\rm{and}}\,{f}_{\gamma }=-\,P\simeq \lambda +\nu ({v}_{\alpha ,\beta }+{v}_{\beta ,\alpha })({v}_{\beta }{\gamma }_{\alpha }{)}_{p},$$

where ${\boldsymbol{\gamma }}\times {\boldsymbol{\tau }}={\bf{n}}$. In particular, the compatible linear forms for the moments and normal interaction forces are given by^11^

39 $${f}_{n}\simeq \overline{k}{\nabla }_{p}[{{\boldsymbol{\tau }}}_{p}\cdot ({\nabla }_{p}^{2}z){{\boldsymbol{\gamma }}}_{p}]\cdot {{\boldsymbol{\tau }}}_{p}-k{{\boldsymbol{\gamma }}}_{p}\cdot {\nabla }_{p}H=\sigma ,$$

where $\tau ^{\prime} (S)=\frac{d\tau }{dS}=\frac{d\tau }{d{\boldsymbol{\theta }}}\cdot \frac{d{\boldsymbol{\theta }}}{dS}={\nabla }_{p}\tau \cdot {{\boldsymbol{\tau }}}_{p}$ and *σ* are the arc length derivative on the projected curve, and the empirical constant accounting for the wetting of the interacting boundary, respectively. Hence, the solution of Eq. (37) can be uniquely determined by imposing the admissible boundary conditions, Eqs. (38) and (39).

### Formulations in the elliptical coordinates

**Figure 1:**
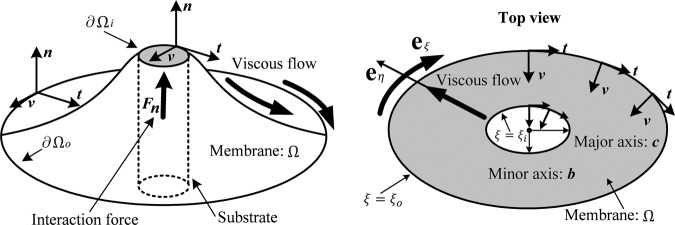
Schematic of an elliptical cylinder substrate-membrane system.

We consider the cases when lipid membranes interact through the elliptical contact domain of a transmembrane substrate, and are subjected a general class of intra-membrane viscous flow (see, Fig. 1). The deformations of lipid membranes defined on an elliptical domain can be examined by using the mapping,

40 $$x+iy=c\,\cosh (\xi +i\eta ),$$

such that

41 $$x=c\,\cosh (\xi )\,\cos (\eta )\,{\rm{and}}\,y=c\,\sinh (\xi )\,\sin (\eta ),$$

through which the rectangular Cartesian coordinates $(x,y)$ are mapped to the elliptical coordinates $(\xi ,\eta )$. The semi focal length $c$ is defined by $c=\sqrt{{a}^{2}-{b}^{2}}$ and $\xi \in [0,\infty )$, and $\eta \in [0,2\pi ]$ are respectively the radial and angular coordinates. Accordingly, Eqs. (40 and 41) furnish the gradient and Laplacian in elliptical coordinates as

42 $$\nabla =\frac{1}{\sqrt{{c}^{2}({\cosh }^{2}\,\xi -{\cos }^{2}\,\eta )}}(\frac{\partial }{\partial \xi }{{\bf{e}}}_{\xi },\frac{\partial }{\partial \eta }{{\bf{e}}}_{\eta }),$$

43 $$\Delta =\frac{1}{{c}^{2}({\cosh }^{2}\,\xi -{\cos }^{2}\,\eta )}(\frac{{\partial }^{2}}{\partial {\xi }^{2}}+\frac{{\partial }^{2}}{\partial {\eta }^{2}}).$$

The condition of incompressibility (i.e. ${v}_{\alpha ,\alpha }=0$) then yields

44 $$\frac{1}{\sqrt{{c}^{2}({\cosh }^{2}\,\xi -{\cos }^{2}\,\eta )}}({v}_{\xi ,\xi }+{v}_{\eta ,\eta })=0,$$

from which the admissible set of viscous flow field is found to be

45 $${v}_{\xi }=\int \,w(\xi ,\eta )d\xi +{C}_{1}\eta \,{\rm{and}}\,{v}_{\eta }=\int \,-\,w(\xi ,\eta )d\eta +{C}_{2}\xi ,$$

so that Eq. (44) is satisfied (i.e. ${v}_{\xi ,\xi }+{v}_{\eta ,\eta }=w(\xi ,\eta )-w(\xi ,\eta )=0$). In the analysis, we assume ${C}_{1}={C}_{2}=0$ for the sake of simplicity. The cases of non-zero coefficients can be easily accommodated via the principles of superposition which will be discussed in the later section.

The membrane-substrate interaction occurs through the wall of the elliptical substrate where the corresponding domain of interest, $\Omega$, and interacting boundary, $\partial \Omega$, are defined respectively as

46 $$\Omega ={\xi }_{i}\le \xi \le {\xi }_{o}({\rm{elliptical}}\,{\rm{annulus}}),\,{\rm{and}}\,\partial \Omega =\xi ={\xi }_{i}\,({\rm{interacting}}\,{\rm{boundary}}).$$

Using the mapping functions in Eqs. (40 and 41), the associated boundary conditions can be obtained from Eq. (38) such that

47 $$\begin{array}{rcl}0 & \simeq  & \frac{\nu }{\sqrt{{c}^{2}({\cosh }^{2}\,\xi -{\cos }^{2}\,\eta )}}({v}_{\alpha ,\beta }+{v}_{\beta ,\alpha })({\tau }_{\beta }{\gamma }_{\alpha }{)}_{p},\,{\rm{and}}\\ -\,\lambda -P & \simeq  & \frac{\nu }{\sqrt{{c}^{2}({\cosh }^{2}\,\xi -{\cos }^{2}\,\eta )}}({v}_{\alpha ,\beta }+{v}_{\beta ,\alpha })({\gamma }_{\beta }{\gamma }_{\alpha }{)}_{p}.\end{array}$$

Here, the repeated indices, $\alpha$ and $\beta$, when summed over their ranges {$1,2$}, refer to $\xi$ and $\eta$ in elliptic coordinates. On the boundaries (i.e. $\xi ={\xi }_{o}$ and $\xi ={\xi }_{i}$), we find

48 $${\boldsymbol{\tau }}={{\bf{e}}}_{\eta }={\rm{and}}\,{\boldsymbol{\gamma }}=-\,{{\bf{e}}}_{\xi },$$

and thereby reduce Eq. (47) to

49 $$\begin{array}{rcl}0 & \simeq  & \frac{\nu }{\sqrt{{c}^{2}({\cosh }^{2}\,\xi -{\cos }^{2}\,\eta )}}({v}_{\xi ,\eta }+{v}_{\eta ,\xi }),\,{\rm{and}}\\ -\,\lambda -P & \simeq  & \frac{\nu }{\sqrt{{c}^{2}({\cosh }^{2}\,\xi -{\cos }^{2}\,\eta )}}\mathrm{(2}{v}_{\xi ,\xi }\mathrm{)}.\end{array}$$

In particular, since the membrane-substrate interaction condition (i.e. ${\bf{n}}-\nabla z={\bf{k}}$, see^11,13^) requires $\nabla z=0$ at the inner boundary ($\xi ={\xi }_{i}$), the normal force (Eq. (39)) becomes

50 $${f}_{n}\simeq -\,k{{\boldsymbol{\gamma }}}_{p}\cdot {\nabla }_{p}H=\sigma .$$

We continue by rewriting ${\nabla }_{p}H$ using Eq. (42) and subsequently reduce Eq. (50) to

51 $$\frac{1}{h(\xi ,\eta )}\frac{\partial H}{\partial \xi }=\frac{\sigma }{k},\,{\rm{on}}\,\partial w({\rm{i}}.{\rm{e}}.\,{\rm{at}}\,\xi ={\xi }_{i}),$$

where,

52 $$h(\xi ,\eta )=\sqrt{{c}^{2}({\cosh }^{2}\,\xi -{\cos }^{2}\,\eta )}.$$

Further, applying the similar schemes as in the above, it is not difficult to show

53 $${z}_{,\alpha \beta }{\nu }_{\alpha ,\beta }=\frac{1}{{h}^{3}(\xi ,\eta )}[{z}_{,\xi \xi }{v}_{\xi ,\xi }+{z}_{,\xi \eta }{v}_{\eta ,\xi }+{z}_{,\xi \eta }{v}_{\xi ,\eta }+{z}_{,\eta \eta }{v}_{\eta ,\eta }\mathrm{]}.$$

Consequently, by combining the above results, we reformulate Eq. (37) and the associated boundary conditions as

54 $$\frac{1}{2}k\Delta (\Delta z)-\lambda \Delta z-\frac{2v}{{h}^{3}(\xi ,\eta )}({z}_{,\xi \xi }{v}_{\xi ,\xi }+{z}_{,\xi \eta }{v}_{\eta ,\xi }+{z}_{,\xi \eta }{v}_{\xi ,\eta }+{z}_{,\eta \eta }{v}_{\eta ,\eta })=\mathrm{0,}$$

subjected to

55 $$z({\xi }_{i},\eta )=0,\,\nabla z({\xi }_{i},\eta )=0\,{\rm{and}}\,\frac{1}{h}\frac{\partial }{\partial \xi }H({\xi }_{i},\eta )=\frac{\sigma }{k}.$$

#### **Remark 1**.

It should be noted that the restrictions on the continuity conditions (${\rm{div}}({\bf{v}})=0$) and the prescribed tangential (${f}_{\tau }=0$) force can be relaxed along and within the elliptical boundaries unlike those arising in circular cases where the admissible set of viscous flows are required to be strictly uniform in one of the coordinate directions (i.e. either ${v}_{r}=const$ or ${v}_{\theta }=const$) to satisfy the constraints^28^. This is mainly due to the confined descriptions of the circular interaction boundary where the rate of change in the unit normal and tangent on the circular boundary remains constant so that the associated normal velocity fields ${v}_{r}$ always points to the center of a circular substrate. Thus, ${v}_{r}$ is required to be vanished by its gradient ${v}_{r,r}$ or gradient of tangential velocity ${v}_{\theta ,\theta }$ to satisfy the continuity condition; i.e.,

56 $${\rm{div}}({\bf{v}})={v}_{r,r}+\frac{{v}_{\theta ,\theta }}{r}+\frac{{v}_{r}}{r}=0.$$

Such restriction can be relaxed in the case of the elliptic interaction boundary, since the rate of change in local coordinate is not necessarily constant, yet they vary with respect to the coordinates $\xi$ and $\eta$ (see, Eqs. (42 and 43)). This further suggests that the normal velocity field ${v}_{\xi }$ does not necessarily points to the center of an elliptical substrate (see, Fig. 1) and therefore no restrictions are necessary for ${v}_{\xi }$. In results, the associated flow fields (${v}_{\xi }$ and ${v}_{\eta }$) can accommodate more general forms such as non-uniform viscous flows and periodic wave form of viscous flows (no need to be strictly constant) without violating the aforementioned constitutive restrictions. Examples regarding these cases will be discussed in the following section.

Lastly, Eqs. (54 and 55) serve as the linearized shape equation system which describes the morphology of lipid membranes under the influences of membrane-substrate interactions and general forms of viscous flows. In the analysis, we also impose $z({\xi }_{i},\eta )=0$ for the purpose of comparison with the existing literature.

## Solutions to the Linearized Systems

It can be seen from Eqs. (42), (43) and (54) that the gradient, Laplacian and the resulting PDEs in elliptical curvilinear coordinate continuously vary with respect to the material points $(\xi ={\xi }_{o},\eta ={\eta }_{o})$ on the membrane surface, where ${\xi }_{o}$ and ${\eta }_{o}$ denote a particular configuration of the surface. In other words, the associated tangential and normal velocities are simultaneously updated as material points move over the membrane surface. Therefore, the solution of the Eq. (54), which is coupled with the viscous velocity fields, cannot be accommodated by the conventional separation variable method of modified Helmholtz equation. In this section, we combine the method of adoptive iteration and the principle of eigenfunction expansions^29–31^, and obtained the complete expression of the membrane’s shape function $z(\xi ,\eta )$.

To proceed, we assume the solution of the form

57 $$z(\xi ,\eta )=\frac{2}{{\mu }^{2}}H(\xi ,\eta )+{B}_{m}+\varphi (\xi ,\eta \mathrm{)}.$$

Here, $\varphi (\xi ,\eta )$ is the plane harmonic function, which is chosen as

58 $$\varphi (\zeta ,\eta )={C}_{m}\,\log ({e}^{\xi }/{e}^{{\xi }_{0}})+{D}_{m}\mathop{\sum ^{\infty }}\limits_{m=0}\,{e}^{-\xi }c{e}_{m}(\eta ,q)$$

to accommodate the desired behavior (i.e. $|\nabla z|\to 0$) as approaching the boundary. In particular, the unknown potential $H(\xi ,\eta ,q)$ can be expressed as^40^

59 $$H(\xi ,\eta )\equiv \mathop{\sum _{m=0}}\limits^{\infty }\,\mathop{\sum ^{\infty }}\limits_{n=0}\,{A}_{m}K{e}_{m}(\xi ,q)c{e}_{n}(\eta ,q){T}_{mn}(\xi ,\eta ),$$

where $c{e}_{n}(\eta ,q)$ and $K{e}_{m}(\xi ,q)$ are the modified Mathieu functions of the first and second kind, respectively, and $q$ ($q > 0$) is the associated parameter (see, for example^41^).

Now, by substituting Eqs. (45) and (59) into Eq. (54), and invoking the orthogonal properties of the Mathieu function,

60 $${\int }_{0}^{2\pi }\,c{e}_{m}(\eta ,q)c{e}_{n}(\eta ,q)d\eta ={\int }_{0}^{2\pi }\,K{e}_{m}(\xi ,q)K{e}_{n}(\xi ,q)d\xi =\pi {\delta }_{mn},\,{\rm{and}}$$

61 $${\int }_{0}^{2\pi }\,{\int }_{0}^{2\pi }\,K{e}_{m}(\xi ,q)c{e}_{n}(\eta ,q)K{e}_{k}(\xi ,q)c{e}_{l}(\eta ,q)d\xi d\eta =\pi {\delta }_{mk},$$

we obtain the following expressions for ${T}_{mn}$:

62 $$\begin{array}{rcl}{T}_{mn}(\xi ,\eta ) & = & {\int }_{0}^{2\pi }\,{\int }_{0}^{2\pi }\,\mathop{\sum _{m=0}}\limits^{\infty }\,\mathop{\sum ^{\infty }}\limits_{n=0}\,-\,\tfrac{\nu }{\lambda {\pi }^{2}{h}^{3}(\xi ,\eta )}\\  &  & \times \,[-{e}^{-\xi }c{e^{\prime} }_{m}(\eta ,q)K{e}_{m}(\xi ,q)c{e}_{n}(\eta ,q)(\tfrac{\partial \,\int \,w(\xi ,\eta )d\xi }{\partial \eta }\\  &  & +\,\tfrac{\partial \,\int \,-w(\xi ,\eta )d\eta }{\partial \xi })+\{{e}^{-\xi }\pi K{e}_{m}(\xi ,q)+{e}^{-\xi }c{e^{\prime\prime} }_{m}(\eta ,q)\\  &  & \times \,K{e}_{m}(\xi ,q)c{e}_{n}(\eta ,q)\}w(\xi ,\eta )]d\xi d\eta .\end{array}$$

The detailed procedures which can be found in^29–31^ are omitted here for the sake of brevity.

Consequently, the general solution in Eq. (57) can be found in the form

63 $$\begin{array}{rcl}z(\xi ,\eta ) & = & \frac{2}{{\mu }^{2}}\,\mathop{\sum _{m=0}}\limits^{\infty }\,[{A}_{m}K{e}_{m}(\xi ,q)c{e}_{m}(\eta ,q){T}_{mn}(\xi ,\eta )\\  &  & +\,{B}_{m}+{C}_{m}\,\log ({e}^{\xi }/{e}^{{\xi }_{0}})+{D}_{m}{e}^{-\xi }c{e}_{m}(\eta ,q)],\end{array}$$

where $\mu =\sqrt{2\lambda /k}$ is the natural length scale which is commonly adopted in the membrane studies (see, for example^11,14,25^). Details regarding the dimensionless variables adopted in the present work will be discussed in later section. The unknown constants ${A}_{m},{B}_{m},{C}_{m}$ and ${D}_{m}$ can be completely determined by imposing the admissible boundary conditions. For instance, the substrate-membrane interaction conditions (55) require

64 $$\frac{\partial }{\partial \xi }z({\xi }_{i},\eta )=0,\,\frac{\partial }{\partial \eta }z({\xi }_{i},\eta )=0,\,z({\xi }_{i},\eta )=0\,{\rm{and}}\,\frac{1}{h({\xi }_{i},\eta )}\frac{\partial }{\partial \xi }H({\xi }_{i},\eta )=\frac{\sigma }{k},$$

on the boundary from which we find that,

$$\begin{array}{rcl}{A}_{m} & = & \mathop{\sum _{m=0}}\limits^{\infty }\,\tfrac{\sigma h({\xi }_{i},\eta )}{k(K{e}_{m}({\xi }_{i},q)c{e}_{m}(\eta ,q){T^{\prime} }_{\xi }({\xi }_{i},\eta {\boldsymbol{)}}+c{e}_{m}(\eta ,q)K{e^{\prime} }_{m}({\xi }_{i},q){T}_{mn}({\xi }_{i},\eta ))},\\ {B}_{m} & = & \mathop{\sum _{m=0}}\limits^{\infty }[\tfrac{2c{e}_{m}(\eta ,q)h({\xi }_{i},\eta )\sigma (K{e}_{m}({\xi }_{i},q)c{e}_{m}(\eta ,q){T^{\prime} }_{\eta }({\xi }_{i},\eta )+K{e}_{m}({\xi }_{i},q)c{e^{\prime} }_{m}(\eta ,q){T}_{mn}({\xi }_{i},n))}{dc{e}_{m}(\eta ,q)k{\mu }^{2}(K{e}_{m}({\xi }_{i},q)c{e}_{m}(\eta ,q){T^{\prime} }_{\xi }(\ell ,\eta )+c{e}_{m}(\eta ,q)K{e^{\prime} }_{m}({\xi }_{i},q){T}_{mn}({\xi }_{i},\eta ))}\\  &  & -\,\tfrac{2\sigma K{e}_{m}({\xi }_{i},q)c{e}_{m}(\eta ,q)h({\xi }_{i},\eta ){T}_{mn}({\xi }_{i},\eta )}{k{\mu }^{2}(K{e}_{m}({\xi }_{i},q)c{e}_{m}(\eta ,q){T^{\prime} }_{\xi }({\xi }_{i},\eta )+c{e}_{m}(\eta ,q)K{e^{\prime} }_{m}({\xi }_{i},q){T}_{mn}({\xi }_{i},\eta ))}].\\ {C}_{m} & = & \mathop{\sum _{m=0}}\limits^{\infty }[-\tfrac{2\sigma h({\xi }_{0},\eta )}{k{\mu }^{2}}\\  &  & -\,\tfrac{2\sigma c{e}_{m}(\eta ,q)h({\xi }_{i},\eta )\{K{e}_{m}({\xi }_{i},q)c{e}_{m}(\eta ,q){T^{\prime} }_{\xi }({\xi }_{i},\eta )+K{e}_{m}({\xi }_{i},q)c{e^{\prime} }_{m}(\eta ,q){T}_{mn}({\xi }_{i},\eta )\}}{c{e^{\prime} }_{m}(\eta ,q)k{\mu }^{2}(K{e}_{m}({\xi }_{i},q)c{e}_{m}(\eta ,q){T^{\prime} }_{\xi }({\xi }_{i},\eta )+c{e}_{m}(\eta ,q)K{e^{\prime} }_{m}({\xi }_{i},q){T}_{mn}({\xi }_{0},\eta ))}],\end{array}$$

and

65 $${D}_{m}=\mathop{\sum _{m=0}}\limits^{\infty }\,-\,\tfrac{2\sigma h({\xi }_{i},\eta ){e}^{{\xi }_{i}}[K{e}_{m}({\xi }_{i},q)c{e}_{m}(\eta ,q){T^{\prime} }_{\eta }({\xi }_{i},\eta )+K{e}_{m}({\xi }_{i},q)c{e^{\prime} }_{m}(\eta ,q){T}_{mn}({\xi }_{i},\eta )]}{c{e^{\prime} }_{m}(\eta ,q)k{\mu }^{2}[K{e}_{m}({\xi }_{i},q)c{e}_{m}(\eta ,q){T^{\prime} }_{\eta }({\xi }_{i},\eta )+c{e}_{m}(\eta ,q)K{e^{\prime} }_{m}({\xi }_{i},q){T}_{mn}({\xi }_{i},\eta )]},$$

where

66 $$\begin{array}{rcl}K{e^{\prime} }_{m}(\xi ,q) & = & \tfrac{\partial K{e}_{m}(\xi ,q)}{\partial \xi },\,c{e^{\prime} }_{m}(\eta ,q)=\tfrac{\partial c{e}_{m}(\eta ,q)}{\partial \eta },\,{T^{\prime} }_{\xi }(\xi ,\eta )=\tfrac{\partial {T}_{mn}(\xi ,\eta )}{\partial \xi },\\ {T^{\prime} }_{\eta }(\xi ,\eta ) & = & \tfrac{\partial {T}_{mn}(\xi ,\eta )}{\partial \eta },{T^{\prime} }_{\xi }({\xi }_{i},\eta )={[\tfrac{\partial {T}_{mn}(\xi ,\eta )}{\partial \xi }]}_{\xi ={\xi }_{i}}\,{\rm{and}}\,{T^{\prime} }_{\eta }({\xi }_{i},\eta )=\tfrac{\partial {T}_{mn}({\xi }_{i},\eta )}{\partial \eta }.\end{array}$$

### Constant viscous flow potential: *w*(*ξ*, *η*) = *A*

The membrane systems may be exposed to the constant viscous flow within a cell such as the selective transport of molecules inside the lipid bilayers where the molecules travel with the constant flow from one side of the membrane to the objective protein^42^. To assimilate such constant viscous flow, we consider the case when $w(\xi ,\eta )=A$, and thereby reduce Eq. (62) to

67 $$\begin{array}{rcl}{T}_{mn}(\xi ,\eta ) & = & {\int }_{0}^{2\pi }\,{\int }_{0}^{2\pi }\,\mathop{\sum _{m=0}}\limits^{\infty }\,\mathop{\sum ^{\infty }}\limits_{n=0}\,-\,\frac{1}{\lambda {\pi }^{2}}\frac{\nu A{e}^{-\xi }K{e}_{m}(\xi ,q)}{{h}^{3}(\xi ,\eta )}\\  &  & \times \,[\pi +\frac{{\partial }^{2}c{e}_{m}(\eta ,q)}{\partial {\eta }^{2}}c{e}_{n}(\eta ,q)]d\xi d\eta .\end{array}$$

Equations (63), (65) and (67) then deliver

68 $$\begin{array}{rcl}z(\xi ,\eta ) & = & \mathop{\sum _{m=0}}\limits^{\infty }\,[\tfrac{2\sigma h(\xi ,\eta )c{e}_{m}(\eta ,q)}{{\sigma }_{1}}\{K{e}_{m}(\xi ,q){T}_{mn}(\xi ,\eta )-K{e}_{m}({\xi }_{0},q){T}_{mn}({\xi }_{0},\eta )\}\\  &  & -\,(\tfrac{2h\sigma }{k{\mu }^{2}}+\tfrac{2\sigma c{e}_{m}(\eta ,q)h(\xi ,\eta )K{e}_{m}({\xi }_{0},q)c{e}_{m}(\eta ,q)\{{T^{\prime} }_{\eta }({\xi }_{0},\eta )+{T}_{mn}({\xi }_{0},\eta )\}}{{\sigma }_{1}c{e^{\prime} }_{m}(\eta ,q),})\,\log (\frac{{e}^{\xi }}{{e}^{{\xi }_{i}}})],\end{array}$$

where

69 $${\sigma }_{1}=k{\mu }^{2}[K{e}_{m}({\xi }_{i},q)c{e}_{m}(\eta ,q)dT\xi ({\xi }_{i},\eta ,q)+c{e}_{m}(\eta ,q)dK{e}_{m}({\xi }_{i},q){T}_{mn}({\xi }_{i},\eta ,q\mathrm{)]}.$$

**Figure 2:**
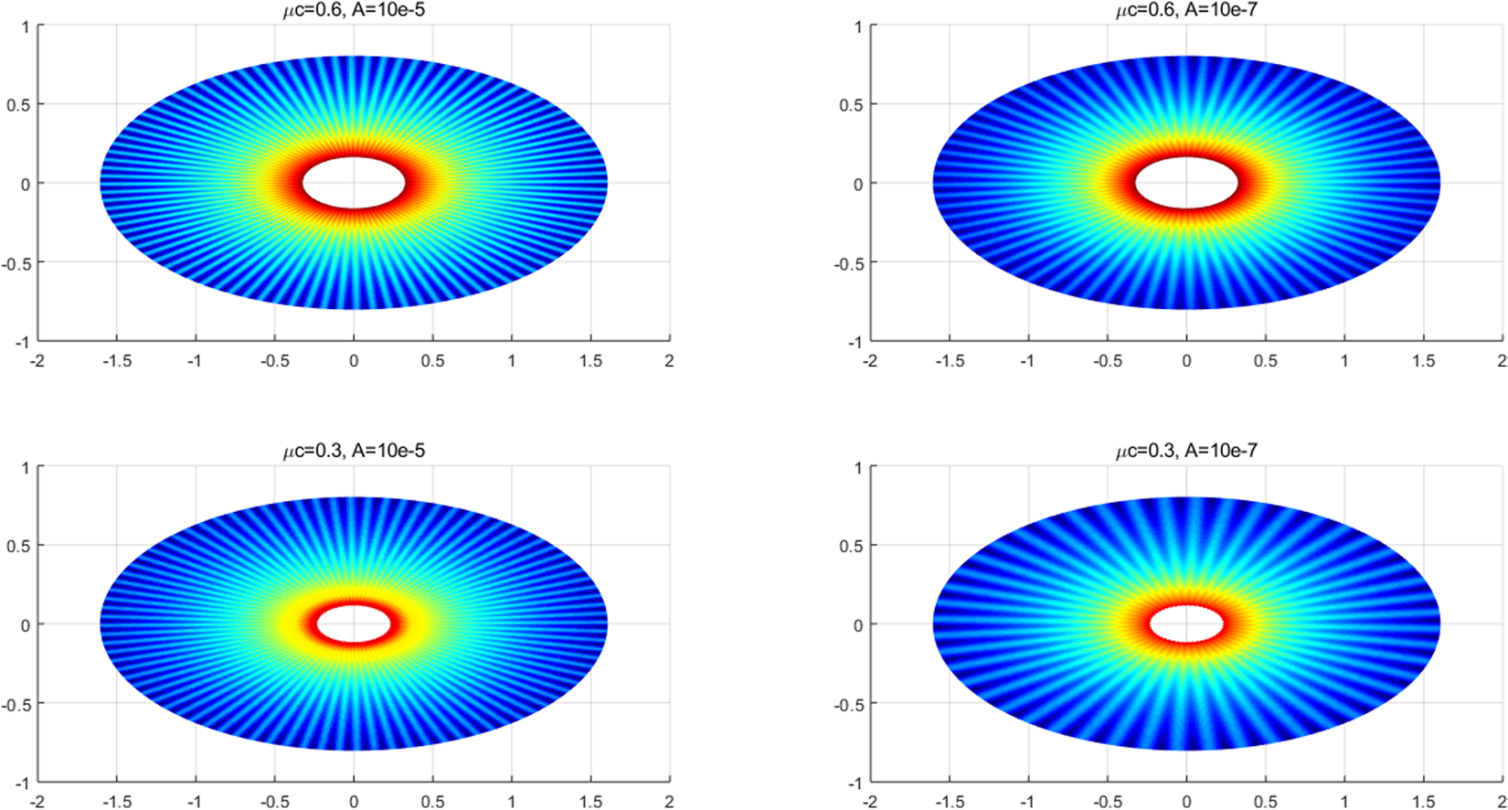
Number of wrinkles with respect to *A* and *μc* (inner radius: major axis).

**Figure 3:**
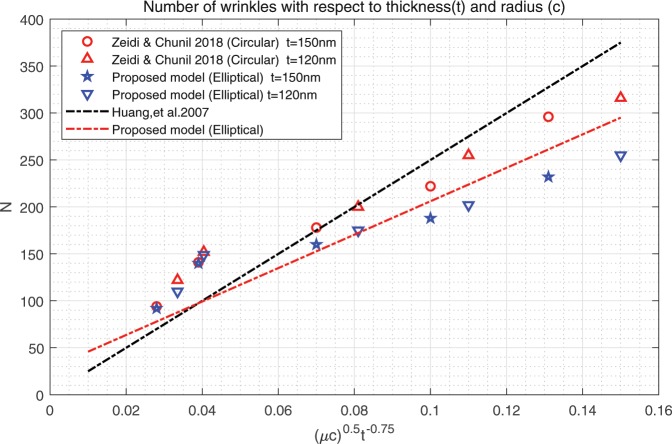
Comparisons: Number of wrinkles on thin polymer films^32^.

The resulting function $z(\xi ,\eta )$ in Eq. (68) describes the morphology of a lipid membrane when subjected to membrane-substrate interactions and the effects of uniform intra-membrane viscous flow. The associated results are presented in Figs. 1, 2 and 3. In the assimilation, we adopt the value of intra-membrane surface viscosity $\nu ={10}^{-4}\,pN\cdot s/nm$ and the flexural modulus of the membrane $k=82\,pN\cdot nm$ from the work of^43,44^. The Lagrange multiplier $\lambda$ is dependent on membrane systems in consideration and usually do not have definite range of values. The values of $\lambda$ commonly used in the literatures is about $\lambda \propto {10}^{-4}\,pN/nm$. In the present study, we assimilate data under the normalized setting using the aforementioned values. The dimensionless parameters used in the simulations are adopted from the works^11,14,25^ as;

70 $$\begin{array}{l}\mu =\sqrt{2\lambda /k}:{\rm{natural}}\,{\rm{length}}\,{\rm{scale}}\,({\rm{e}}.\,{\rm{g}}.\,\mu a\,:\,{\rm{radius}}\,{\rm{of}}\,{\rm{a}}\,{\rm{circular}}\,{\rm{membrane}}),\\ \sigma /\lambda :{\rm{force}}\,{\rm{scale}}\,({\rm{e}}{\rm{.g}}.\,{f}_{n}=\sigma /\lambda \,:\,{\rm{interaction}}\,{\rm{force}}).\end{array}$$

**Figure 4:**
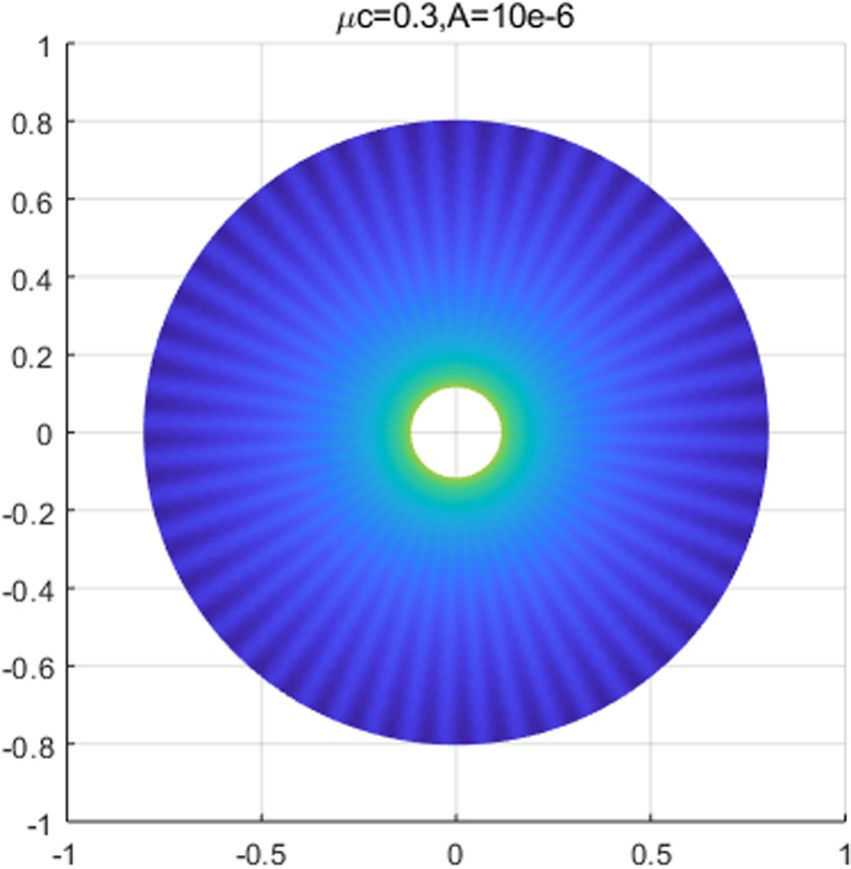
Comparison with circular case^28^ (Fig. 5) (47 wrinkles in total).

We have found that the viscous flow gives rise to wrinkle phenomena, when the normalized magnitude of viscous flow is greater than the critical number (i.e. $\tfrac{A\nu }{\lambda }\ge {10}^{-15}$). Especially, the number of radial wrinkles with respect to the intra-membrane viscous flows (*A*) and the radius of the inner ellipse (*μc*) are illustrated in Fig. 2. The top two figures indicate that, with the same inner ellipse, the number of wrinkles reduce as the magnitude of viscous flow decreases from ${10}^{-5}$ to ${10}^{-7}$. Further, the right and/or left two figures shows that the number of wrinkles increase as the inner radius of an ellipse increase from 0.3 to 0.6 while the magnitude of the viscous flow remains the same (i.e. $A={10}^{-5}$ (left) and $A={10}^{-7}$ (right)). Phenomenologically compatible results can be found in the relevant works such as circular substrate-membrane interactions^28^, capillary wrinkles on thin polymer films^32^ and theoretical study on an elastic surface^45^, where the number of radial wrinkles depends upon the size of the inner radius and membrane thickness. The proposed model successfully reproduces the reported results under the physically similar/compatible settings (see Figs. 3 and 4). In fact, the solutions presented in^28^ are the special case of the presented solution (see, Figs. 3 and 4) in the limit of vanishing eccentricity of elliptical domains (i.e. $h({\xi }_{i},\eta )=a{(1-{e}^{2}{\cos }^{2}\eta )}^{1/2}=a$ for $e\to 0$). Lastly, we note here that the predicted wrinkle states are unique and stable, since the proposed model satisfies strict quasi-convexity via the minimization of membranes’ strain-energy potentials (see, for example^45,46^).

### Non-uniform viscous flow potential: *w*(*ξ*, *η*) = *A* sin *ξ* cos *η* (waveform)

In this section, we consider membrane systems with non-uniform viscous flow. The non-uniform cases can be observed in various cellular activities such as the transportation of the intracellular membrane and the transmembrane proteins induced by the viscous flow with tension gradient^47^. In this case, the viscose flow field becomes non-uniform due to the interactions with tension gradient field.

Membranes subjected to the waveform of non-uniform viscous flows can be examined by introducing the following potential function,

71 $$w(\xi ,\eta )=A\,\sin (E\xi )\,\cos (F\eta ),$$

where the intensity of wavy flow can be controlled by the parameters $E$ and $F$. In the assimilation, we set $E=F=1$ for simplicity. Accordingly, from Eq. (62), we obtain the following expression of ${T}_{mn}(\xi ,\eta )$, addressing the viscous effects,

72 $$\begin{array}{rcl}{T}_{mn}(\xi ,\eta ) & = & {\int }_{0}^{2\pi }\,{\int }_{0}^{2\pi }\,\mathop{\sum _{m=0}}\limits^{\infty }\,\mathop{\sum ^{\infty }}\limits_{n=0}\,-\,\tfrac{1}{{\pi }^{2}\lambda }\tfrac{\nu A\,\sin \,\xi \,\cos \,\eta {e}^{-\xi }K{e}_{m}(\xi ,q)}{{h}^{3}(\xi ,\eta )}\\  &  & \times \,[\pi +\tfrac{{\partial }^{2}c{e}_{m}(\eta ,q)}{\partial {\eta }^{2}}c{e}_{n}(\eta ,q)]d\xi d\eta .\end{array}$$

**Figure 5:**
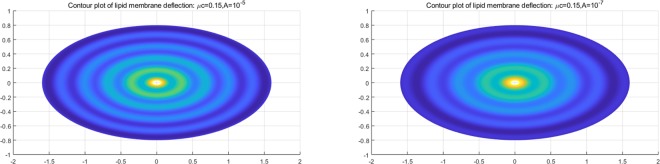
Wave deformations of lipid membrane with respect to *A*.

**Figure 6:**
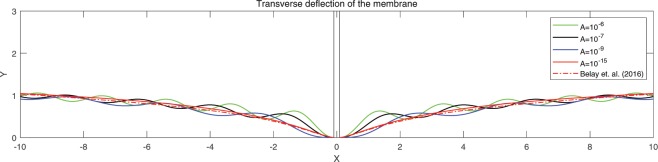
Transverse deflections of lipid membrane with respect to intra-membrane viscous flows.

**Figure 7:**
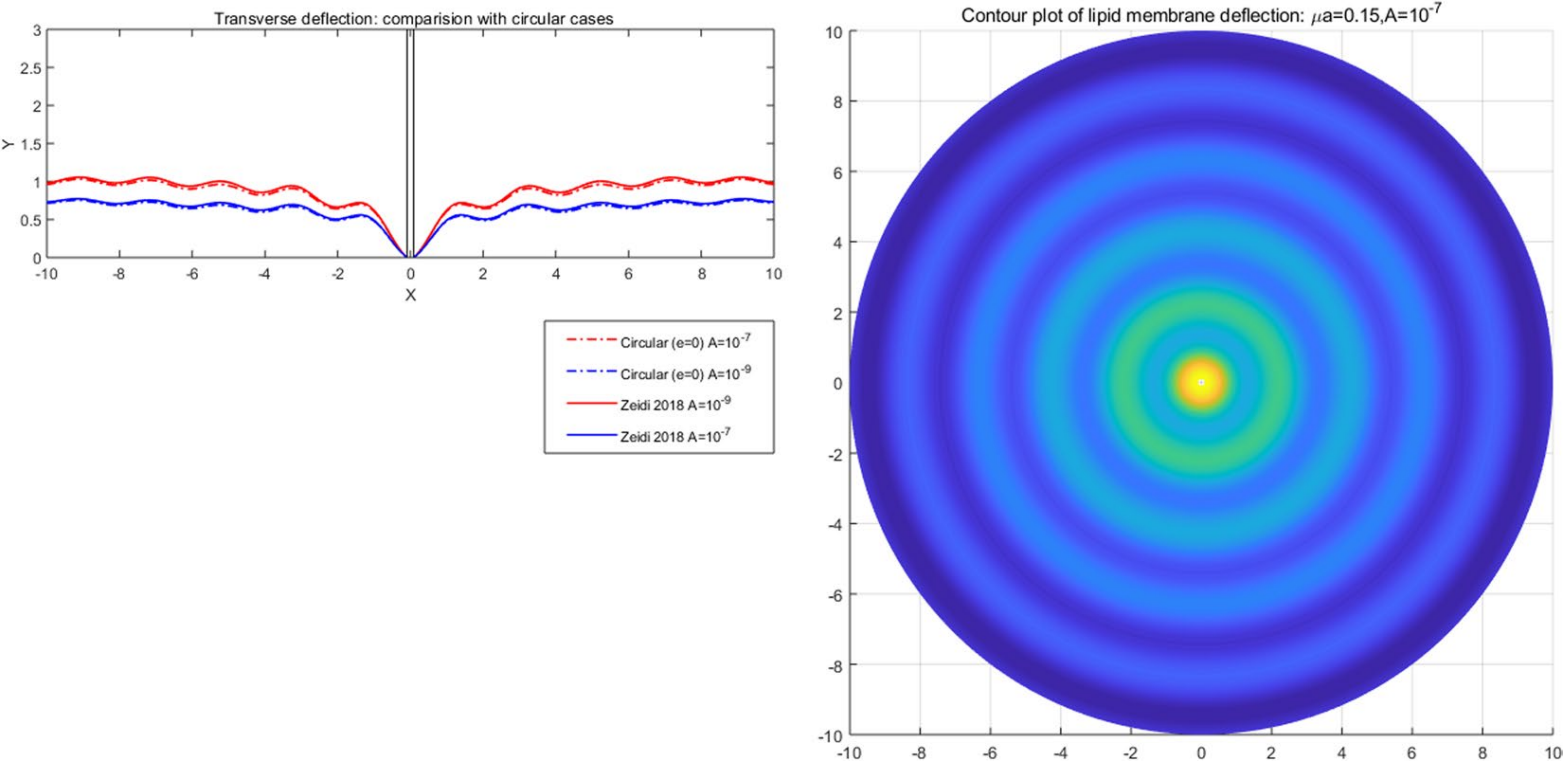
Comparison with circular case^28^ (Figs. 3 and 4).

Combining (63), (65) and (72), the complete solution describing the membranes’ morphology can then be found as

73 $$\begin{array}{rcl}z(\xi ,\eta ) & = & \mathop{\sum _{m=0}}\limits^{\infty }\,[\tfrac{2h\sigma c{e}_{m}(\eta ,q)}{{\sigma }_{1}}\{K{e}_{m}(\xi ,q){T}_{mn}(\xi ,\eta )-K{e}_{m}({\xi }_{i},q){T}_{mn}({\xi }_{i},\eta )\}\\  &  & -\,(\tfrac{2h\sigma }{k{\mu }^{2}}+\tfrac{2\sigma c{e}_{m}(\eta ,q)h(\xi ,\eta )K{e}_{m}({\xi }_{i},q)[c{e}_{m}(\eta ,q){T^{\prime} }_{\eta }({\xi }_{i},\eta )+c{e^{\prime} }_{m}(\eta ,q){T}_{mn}({\xi }_{i},\eta )]}{{\sigma }_{1}dc{e}_{m}(\eta ,q)})\,\log (\tfrac{{e}^{\xi }}{{e}^{{\xi }_{i}}})],\end{array}$$

where ${\sigma }_{1}$ is defined in Eq. (69). Similar to the constant viscous cases, the resulting deformation fields (radial wave deformations) are sensitive to both the dimension of an inner ellipse and the intensity of viscous flow; i.e., the number of waves reduces as *A* decreases from ${10}^{-5}$ to ${10}^{-7}$ (See. Fig. 5). But, more importantly, the transverse wave deformations of the membrane and the corresponding vertical deflections die out as they approach the remote boundary. As a result, the corresponding boundary remains intact and stable (See. Fig. 6). In the event of vanishing $A$, the wave deformations are completely removed from the entire domain of interest so that the vertical deformation profile reduces to the results in^17^, where the authors present the analysis of elliptical substrate-membrane interaction problems without the considerations of viscosity effects (See. Fig. 6). Also, Fig. 7. shows that the obtained solution accommodates the results of circular substrate-membrane interaction problems in^28^ when the eccentricity converges to zero (i.e. $e\to 0$). In fact, the solutions in Figs. 6 and 7 become essentially identical for sufficiently small value of $A$; i.e., $A\le {10}^{-16}$ for case in Fig. 6 and $A\le {10}^{-8}$ and $A\le {10}^{-10}$ for the cases in Fig. 7. In the assimilations, the classical solutions obtained from the proposed model are intentionally reproduced at $A={10}^{-15}$, $A={10}^{-7}$ and $A={10}^{-9}$ for the purpose of visual demonstration.

### Dual source problems: *w*(*ξ*, *η*) = *A* + *A* sin *ξ* cos *η*

The proposed model is sufficiently general in that the viscous effects from both radial and circumferential directions can be simultaneously considered. To demonstrate this, we introduce the following dual source potential

74 $$w(\xi ,\eta )=A+A\,\sin \,\xi \,\cos \,\eta ,$$

and subsequently obtain from Eq. (62) that

75 $$\begin{array}{rcl}{T}_{mn}(\xi ,\eta ) & = & {\int }_{0}^{2\pi }\,{\int }_{0}^{2\pi }\,\mathop{\sum _{m=0}}\limits^{\infty }\,\mathop{\sum ^{\infty }}\limits_{n=0}\,-\,\frac{1}{\lambda {\pi }^{2}}\frac{\nu A\mathrm{(1}+\,\sin \,\xi \,\cos \,\eta ){e}^{-\xi }K{e}_{m}(\xi ,q)}{{h}^{3}(\xi ,\eta )}\\  &  & \times \,[\pi +{e}^{-\xi }\frac{{\partial }^{2}c{e}_{m}(\eta ,q)}{\partial {\eta }^{2}}c{e}_{n}(\eta ,q)]d\xi d\eta .\end{array}$$

Thus, from Eqs. (63) and (65), the deformation mapping function $z(\xi ,\eta )$ can be obtained in the same manner as in the single source cases.

**Figure 8:**
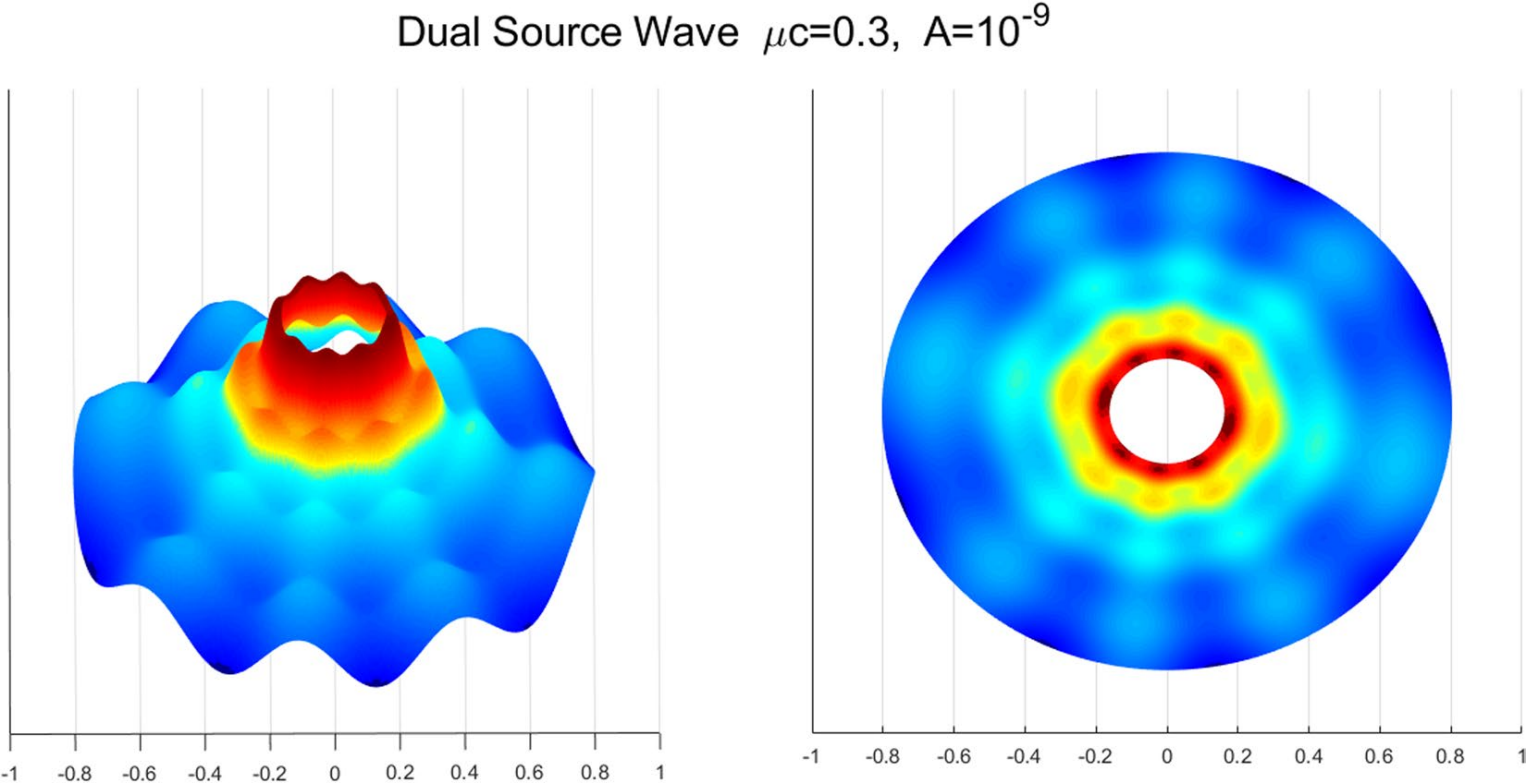
Membrane shape evolutions with dual source viscous effects: *w*(*ξ*; *η*) = *A* + *A* sin *ξ* cos *η*.

**Figure 9:**
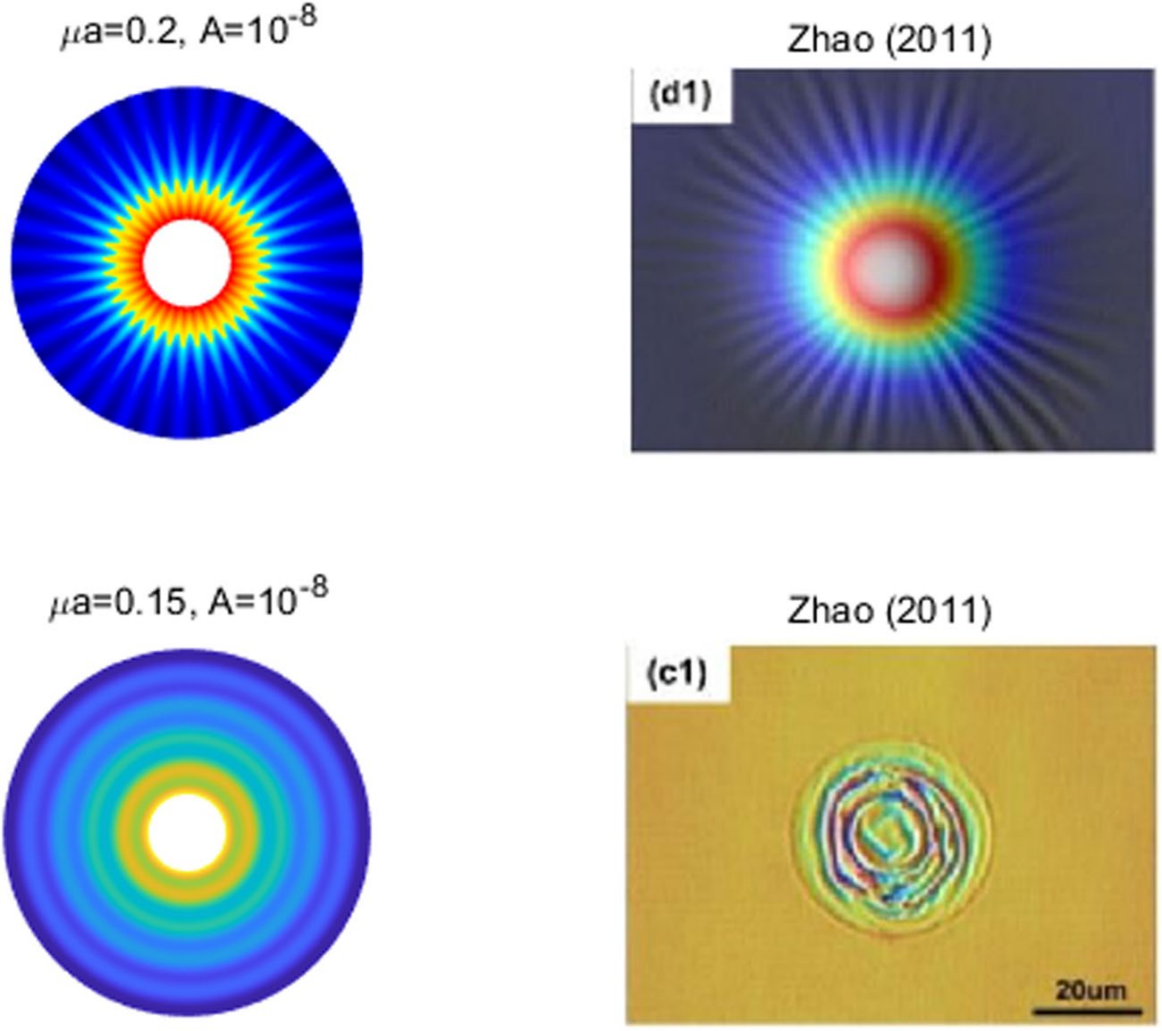
Case study (Single source problem): experimental results in^33^ (Fig. 2). © IOP Publishing. Reproduced with permission. All rights reserved.

**Figure 10:**
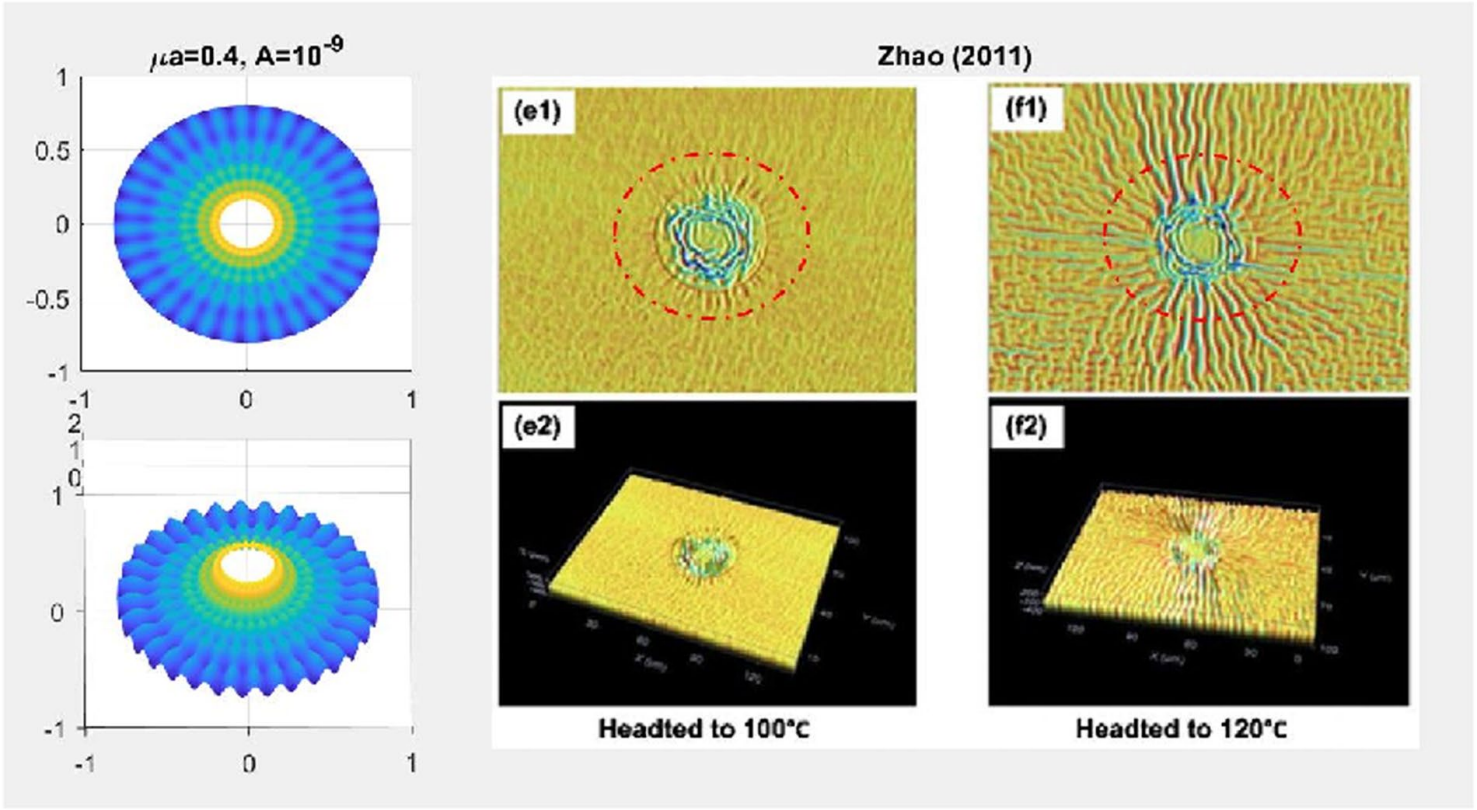
Case study (Dual source problem): experimental results in^33^ (Fig. 3). © IOP Publishing. Reproduced with permission. All rights reserved.

Figure 8 illustrates the deformation configuration of the membranes under the influence of dual source viscous flow. It is shown that both the radial and circumferential wave patterns are simultaneously observed. Morphologically similar cases are reported in the work of^33^ where the authors examined the wrinkle phenomena of a thin gold layer (10 *nm* in thickness) when subjected to thermal stresses from the adjoined polymer substrate. In cases of thin membranes, thermal stresses may be understood as a particular type of the surface stress^48,49^. Therefore, the results may bear close resemblance with the present case where the membrane’s deformations are induced by the surface interaction forces which are transmitted from the acting viscous flows. The obtained solution assimilates the experimental results in^33^ when compatible conditions are applied (see, Figs. 9 and 10). This further suggests that the proposed model may be of practical interest in the morphological study of thin film structures. Such investigations are, however, limited in the present study due to the lack of available data.

#### **Remark 2**.

The results in Fig. 8 further indicates that the principle of superposition remains valid in the present cases. The principle is widely adopted in various engineering problems with simple initial and/or boundary value problems of either first (Dirichlet) or second (Neumann) type^50–52^. However, such practices are largely absent in the membrane studies due the complexity of mixed boundary conditions (i.e. both the Dirichlet and Neumann boundary conditions are prescribed on the boundaries), and the limited access for the solutions of membrane systems subjected to coupled-physics environment. In the present case, the solutions of single source problems (i.e. $w=A$ and $w=A\,\sin \,\xi \,\cos \,\eta$) can be obtained from Eqs. (67), (68), (72) and (73) that

76 $$\begin{array}{rcl}{T}_{mn}(\xi ,\eta ) & = & {\int }_{0}^{2\pi }\,{\int }_{0}^{2\pi }\,\mathop{\sum _{m=0}}\limits^{\infty }\,\mathop{\sum ^{\infty }}\limits_{n=0}\,-\,\frac{1}{\lambda {\pi }^{2}}\frac{\nu A{e}^{-\xi }K{e}_{m}(\xi ,q)}{{h}^{3}(\xi ,\eta )}\\  &  & \times \,[\pi +\frac{{\partial }^{2}c{e}_{m}(\eta ,q)}{\partial {\eta }^{2}}c{e}_{n}(\eta ,q)]d\xi d\eta ,\end{array}$$

77 $$\begin{array}{rcl}z(\xi ,\eta ) & = & \mathop{\sum _{m=0}}\limits^{\infty }\,[\tfrac{2\sigma h(\xi ,\eta )c{e}_{m}(\eta ,q)}{{\sigma }_{1}}\{K{e}_{m}(\xi ,q){T}_{mn}(\xi ,\eta )\\  &  & -\,K{e}_{m}({\xi }_{0},q){T}_{mn}({\xi }_{0},\eta )\}\\  &  & -\,(\tfrac{2h\sigma }{k{\mu }^{2}}+\tfrac{2\sigma c{e}_{m}(\eta ,q)h(\xi ,\eta )K{e}_{m}({\xi }_{0},q)c{e}_{m}(\eta ,q)\{{T^{\prime} }_{\eta }({\xi }_{0},\eta )+{T}_{mn}({\xi }_{0},\eta )\}}{{\sigma }_{1}c{e^{\prime} }_{m}(\eta ,q),})\,\log (\tfrac{{e}^{\xi }}{{e}^{{\xi }_{i}}})],\end{array}$$

for uniform flow $w=A$ and

78 $$\begin{array}{rcl}{T}_{mn}(\xi ,\eta ) & = & {\int }_{0}^{2\pi }\,{\int }_{0}^{2\pi }\,\mathop{\sum _{m=0}}\limits^{\infty }\,\mathop{\sum ^{\infty }}\limits_{n=0}\,-\,\frac{1}{{\pi }^{2}\lambda }\frac{\nu A\,\sin \,\xi \,\cos \,\eta {e}^{-\xi }K{e}_{m}(\xi ,q)}{{h}^{3}(\xi ,\eta )}\\  &  & \times \,[\pi +\frac{{\partial }^{2}c{e}_{m}(\eta ,q)}{\partial {\eta }^{2}}c{e}_{n}(\eta ,q)]d\xi d\eta ,\end{array}$$

79 $$\begin{array}{rcl}z(\xi ,\eta ) & = & \mathop{\sum _{m=0}}\limits^{\infty }\,[\tfrac{2h\sigma c{e}_{m}(\eta ,q)}{{\sigma }_{1}}\{K{e}_{m}(\xi ,q){T}_{mn}(\xi ,\eta )\\  &  & -\,K{e}_{m}({\xi }_{i},q){T}_{mn}({\xi }_{i},\eta )\}\\  &  & -\,(\tfrac{2h\sigma }{k{\mu }^{2}}+\tfrac{2\sigma c{e}_{m}(\eta ,q)h(\xi ,\eta )K{e}_{m}({\xi }_{i},q)[c{e}_{m}(\eta ,q){T^{\prime} }_{\eta }({\xi }_{i},\eta )+c{e^{\prime} }_{m}(\eta ,q){T}_{mn}({\xi }_{i},\eta )]}{{\sigma }_{1}dc{e}_{m}(\eta ,q)})\,\log (\tfrac{{e}^{\xi }}{{e}^{{\xi }_{i}}})],\end{array}$$

for non-uniform flow $w=A\,\sin \,\xi \,\cos \,\eta$. It is clear from Eqs. (77)–(79) that the structure of the solution $z(\xi ,\eta )$ remains intact. In fact, only ${T}_{mn}(\xi ,\eta )$ part of the solutions (i.e. Eqs. (76) and (78)) are affected with respect to the varying viscous flows. In particular, by adding Eqs. (76) and (78), we find

80 $$\begin{array}{rcl}{T}_{mn}(\xi ,\eta ) & = & {\int }_{0}^{2\pi }\,{\int }_{0}^{2\pi }\,\mathop{\sum _{m=0}}\limits^{\infty }\,\mathop{\sum ^{\infty }}\limits_{n=0}\,-\,\tfrac{1}{\lambda {\pi }^{2}}\tfrac{\nu A\mathrm{(1}+\,\sin \,\xi \,\cos \,\eta ){e}^{-\xi }K{e}_{m}(\xi ,q)}{{h}^{3}(\xi ,\eta )}\\  &  & \times \,[\pi +{e}^{-\xi }\tfrac{{\partial }^{2}c{e}_{m}(\eta ,q)}{\partial {\eta }^{2}}c{e}_{n}(\eta ,q)]d\xi d\eta .\end{array}$$

**Figure 11:**
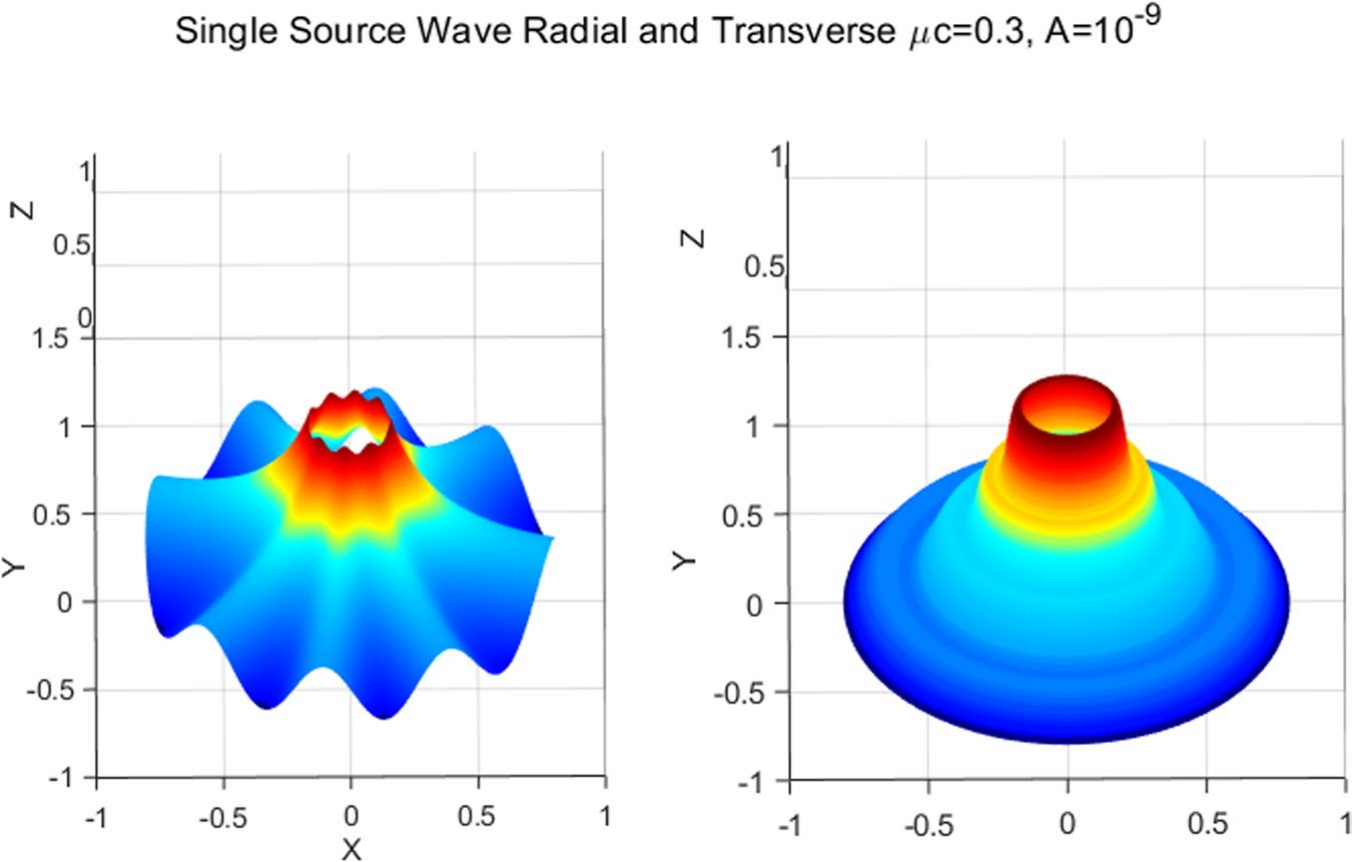
Decomposed solutions of the shape evolution: *w* = *A* (Left); *w* = *A* sin *ξ* cos *η* (Right).

The above is the same as the solution obtained from the dual source problem (Eq. (75) and Fig. 8). This, in turn, suggests that the solutions of dual source problems can be obtained directly from the solutions of single source problems (see, also, Figs. 8 and 11) via simple summations. In other words, the principle of superposition remains valid even with the presence of intra-membrane viscous flows and interaction forces. The result may further promote the study of various different influences of viscous flows onto membrane-substrate systems by minimizing computational complexities and resources.

### Reduction to the circular lipid membrane problems

The solution of a classical membrane-substrate problem^11^ can also be obtained directly from the present model. To demonstrate this, we evaluate (when $e=0$)

81 $$\begin{array}{rcl}h({\xi }_{i},\eta ) & = & a{\mathrm{(1}-{e}^{2}{\cos }^{2}\eta )}^{\frac{1}{2}}=a,c{e}_{m}(\eta ,q)=\frac{1}{\sqrt{2}}({\rm{for}}\,m=0),\\ c{e}_{m}(\eta ,q) & = & \cos \,m\theta ({\rm{for}}\,m\ne 0),K{e}_{m}(\eta ,q)={G}_{m}{K}_{m}(\mu r)\,{\rm{and}}\\ K{e^{\prime} }_{m}(\eta ,q) & = & \mu {G}_{m}{K^{\prime} }_{m}(\mu r),\end{array}$$

where *a*, *m*, and ${K}_{m}(\mu \rho )$ are the radius of the inner circle, the separation constant and the modified Bessel function of second kind of order $m$, respectively. Also, $r=c{e}^{\xi }/2$ and ${G}_{m}$ are arbitrary constants with respect to the order $m$.

Now, substituting the above into Eq. (62) yields

82 $$\begin{array}{rcl}{T}_{mn}(\xi ,\eta ) & = & {T}_{m}(\mu a)={G}_{m}\frac{\nu }{{\pi }^{2}\lambda }\frac{1}{{a}^{3}}\\  &  & \times \,[-\pi \frac{1}{a}{K}_{m}(\mu r)+\frac{1}{a}{K}_{m}(\mu r)]\equiv \frac{1}{a}{\bf{A}}{G}_{m},\end{array}$$

where we define ${\bf{A}}=\frac{\nu }{{\pi }^{2}\lambda }\frac{1}{{a}^{3}}{K}_{m}(\mu r)[1-\pi ].$ Thus, from Eqs. (68) and (82) we find

83 $$\begin{array}{rcl}z(\xi ,\eta )=z(r) & = & \frac{2\sigma a{G}_{m}}{k{\mu }^{3}{G}_{m}{K^{\prime} }_{m}(\mu a)}[{K}_{m}(\mu r)-{K}_{m}(\mu a)]\\  &  & -\,(\frac{2\sigma }{k{\mu }^{2}}+\frac{2\sigma {K}_{m}(\mu a){G}_{m}}{k{\mu }^{3}\frac{1}{{\rho }_{0}}{G}_{m}{K^{\prime} }_{m}(\mu a)})\,\log (\frac{r}{a}).\end{array}$$

But, since ${G}_{m}/{G}_{m}=1$, Eq. (82) further reduces to

84 $$z(r)=\frac{2\sigma }{k{\mu }^{2}}[(\frac{{K}_{m}(\mu r)-{K}_{m}(\mu a)}{\mu {K^{\prime} }_{m}(\mu a)})a-(1+\frac{{K}_{m}(\mu a)a}{\mu {K^{\prime} }_{m}(\mu a)})\,\log (\frac{r}{a})].$$

Finally, we substitute ${\mu }^{2}=2\lambda /k$ in the above and thereby obtain

85 $$z(r)=\frac{2\sigma a}{k{\mu }^{3}{K^{\prime} }_{0}(\mu a)}[{K}_{0}(\mu r)-{K}_{0}(\mu a)]-\frac{\sigma a}{\lambda }(1+\frac{{K}_{0}(\mu a)}{\mu {K^{\prime} }_{0}(\mu a)})\,\log (\frac{r}{a}).$$

The obtained solution in Eq. (85) is the same as in^11^ (Eq. 135), except the Bessel terms associated with the logarithmic function $({\rm{i}}{\rm{.e}}\tfrac{{K}_{0}(\mu a)}{\mu {K^{\prime} }_{0}(\mu a)}\,\log (\tfrac{r}{a}))$.

#### **Remark 3**.

**Figure 12:**
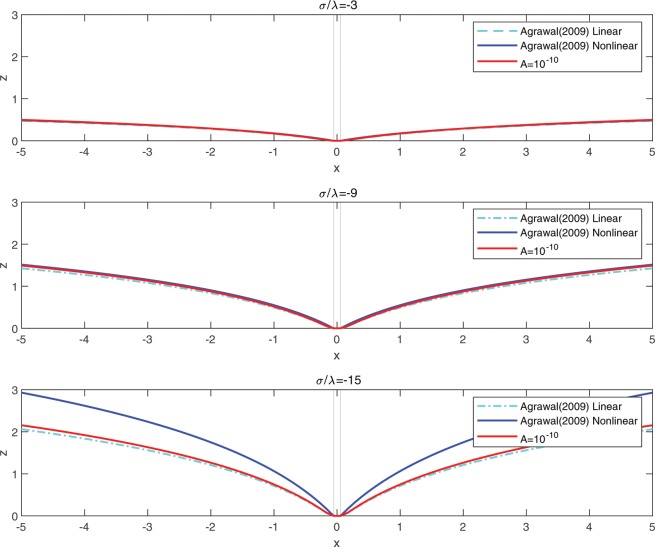
Comparison with existing models: membrane-circular substrate interaction problem^11^.

The method proposed in the present study is unique in that it utilizes both the iterative reduction scheme and the method of eigenfunction expansions while invoking the orthogonal properties of the Mathieu function. This further allows one to identify more wide class of potential functions of Mathieu type that the traditional method is limited in prediction. Further, since the Mathieu potential reduces to the Bessel function at the particular configuration of $e=0$; i.e.,

86 $$K{e}_{m}(\eta ,q)={G}_{m}{K}_{m}(\mu r)\,{\rm{and}}\,K{e^{\prime} }_{m}(\eta ,q)=\mu {G}_{m}{K^{\prime} }_{m}(\mu r)\,{\rm{for}}\,e=0,$$

the solutions of circular substrate interaction problems^11,18^ can be accommodated by the proposed model as a special case (i.e. $e=0$, see, Figs. 7 and 12). In fact, Eq. (85) yields better predictions (slightly more resemble to the non-linear solution) when compared with the existing results (see, Fig. 12). This is due the presence of additionally predicted Bessel terms which cannot be obtained by the classical Helmholtz equations defined in the circular system. This further allows one to consider more general, and perhaps more realistic classes of viscous flows especially those arising in circular boundaries. For example, in circular problems, the generalization of the viscous potential Eq. (45) and the implementation of dual source flow Eq. (74) is no longer possible due to the confined descriptions of the associated circular boundary (see. Remark. 1.). Such difficulties can be overcome by creating desired forms of viscous flows in an elliptic coordinate where the corresponding continuity equation is the same form as in the Cartesian coordinate (i.e. ${v}_{\xi ,\xi }+{v}_{\eta ,\eta }=0$, see, Eq. (44)), and reducing the obtained solutions to the circular cases where the transition is always possible since the conformal mapping of an ellipse to a circle exists^53^.

The results obtained in the present study are of more practical interest in that, when used in conjunction with the principle of superposition (see. Remark. 2.), they essentially lead to the solution of a class of problems in which the viscous effects are characterized by a much wider and more realistic class of functions. Potential applications may be extended to retina clinical study of wrinkle-caused vision impairment^34^ and the effects of viscous flows on essential cellular functions such as fusion, fission and vesicle formation^35,54,55^. For example, the idiopathic epiretinal membranes (iERMs) is a common pathology which have been observed in more than 20% of eyes from elderly person^34,56^. When iERMs are thicker with contractile properties, they cause surface wrinkling of the retina resulting impaired vision (a macular epiretinal membrane)^34,57^. Such wrinkle formations are most often induced by the interactions between the posterior vitreous cortex and the retina^58^. Since nearly all the emmetropic retinas are oblate in shape in both transverse axial and sagittal sections^59^, the wrinkle formations on the retina may share close similarity to the elliptical membrane-substrate systems examined by the proposed model. In addition, wrinkle involved deformations and the directional elongation of the vesicle are often caused by the viscous shear flows and/or directional viscous flows^54,55^. Therefore, the proposed model may be employed to study the morphological transitions of cell membranes associated with those cellular activities.

### Ethics statement

This work did not involve any collection of human data.

## Data acquisition

Figures in the manuscript are prepared by visualizing analytical solutions presented in the manuscript. For the purpose, a commercial software (MATLAB) is used.

## Data Availability

This work does not have any experimental data.
